# A Repurposed Drug Interferes with Nucleic Acid to Inhibit the Dual Activities of Coronavirus Nsp13

**DOI:** 10.1021/acschembio.4c00244

**Published:** 2024-07-09

**Authors:** Nathan Soper, Isabelle Yardumian, Eric Chen, Chao Yang, Samantha Ciervo, Aaron L. Oom, Ludovic Desvignes, Mark J. Mulligan, Yingkai Zhang, Tania J. Lupoli

**Affiliations:** † Department of Chemistry, New York University, New York, New York 10003, United States; ‡ NYU Langone Vaccine Center, Department of Medicine, New York University Grossman School of Medicine, New York, New York 10016, United States; § High Containment Laboratories, Office of Science and Research, NYU Langone Health, New York, New York 10016, United States; ∥ Simons Center for Computational Physical Chemistry at New York University, New York, New York 10003, United States

## Abstract

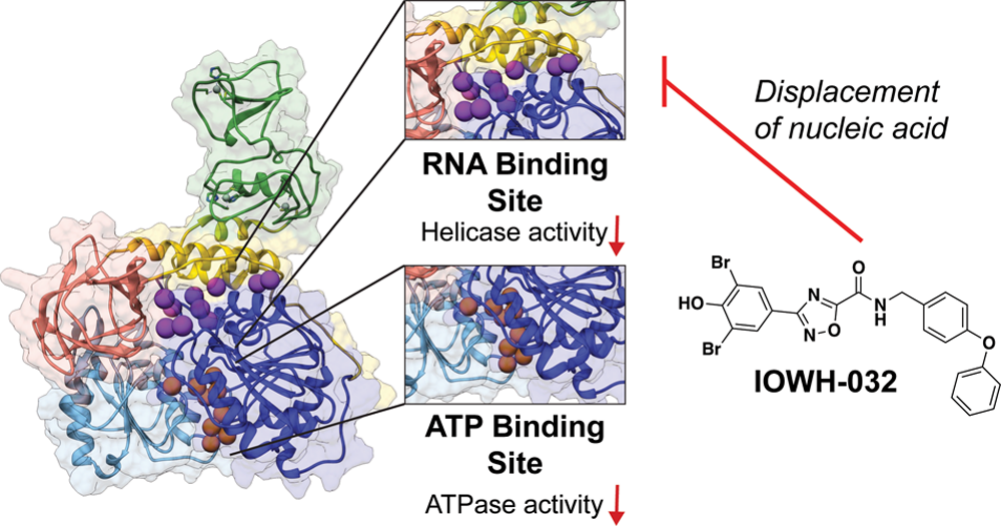

The recent pandemic caused by severe acute respiratory syndrome coronavirus 2 (SARS-CoV-2) highlighted a critical need to discover more effective antivirals. While therapeutics for SARS-CoV-2 exist, its nonstructural protein 13 (Nsp13) remains a clinically untapped target. Nsp13 is a helicase responsible for unwinding double-stranded RNA during viral replication and is essential for propagation. Like other helicases, Nsp13 has two active sites: a nucleotide binding site that hydrolyzes nucleoside triphosphates (NTPs) and a nucleic acid binding channel that unwinds double-stranded RNA or DNA. Targeting viral helicases with small molecules, as well as the identification of ligand binding pockets, have been ongoing challenges, partly due to the flexible nature of these proteins. Here, we use a virtual screen to identify ligands of Nsp13 from a collection of clinically used drugs. We find that a known ion channel inhibitor, IOWH-032, inhibits the dual ATPase and helicase activities of SARS-CoV-2 Nsp13 at low micromolar concentrations. Kinetic and binding assays, along with computational and mutational analyses, indicate that IOWH-032 interacts with the RNA binding interface, leading to displacement of nucleic acid substrate, but not bound ATP. Evaluation of IOWH-032 with microbial helicases from other superfamilies reveals that it is selective for coronavirus Nsp13. Furthermore, it remains active against mutants representative of observed SARS-CoV-2 variants. Overall, this work provides a new inhibitor for Nsp13 and provides a rationale for a recent observation that IOWH-032 lowers SARS-CoV-2 viral loads in human cells, setting the stage for the discovery of other potent viral helicase modulators.

## Introduction

**Figure 1:**
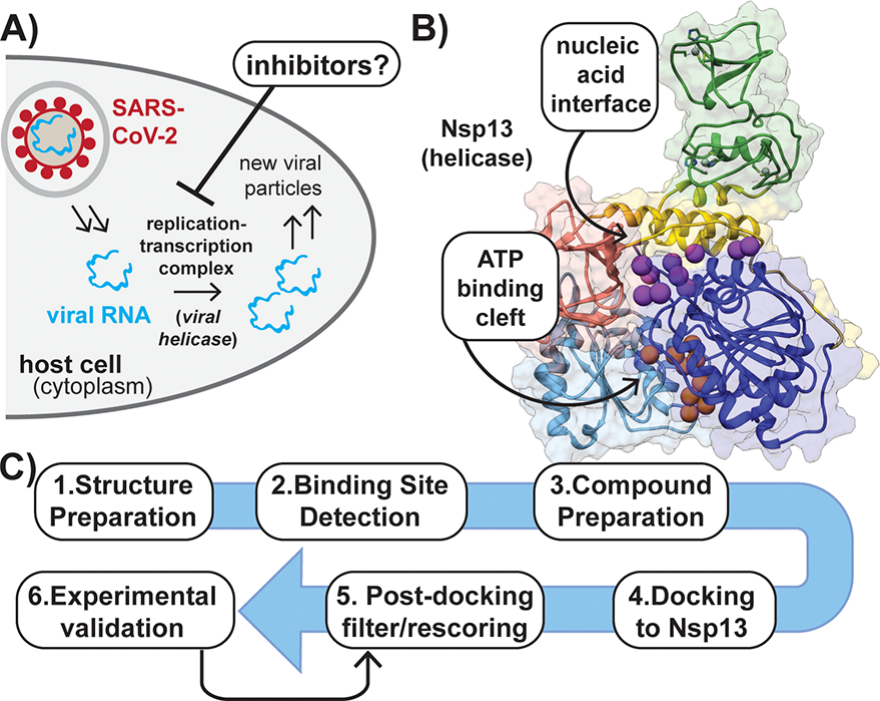
Helicase inhibition is a target for blocking viral replication. (A) Simplified overview of SARS-CoV-2 viral replication in the host. (B) Structure of helicase with the location of the RNA and ATP binding sites indicated by purple and orange balls, respectively. Five domains are shown: zinc-binding domain (green); stalk (yellow); subdomain 1B (salmon); subdomain 1A (dark blue); subdomain 2A (light blue). Gray balls represent bound Zn^2+^. (PDB ID: 6ZSL). (C) Flowchart describing steps employed in virtual screen for Nsp13 inhibitors.

Across all domains of life, helicases use the power of nucleotide hydrolysis to power not only the unwinding of DNA but also the alteration of RNA structure. In cells, helicases unravel RNA during transcription, translation, and splicing, and displace proteins during nucleic acid metabolism.^1^ Similarly, RNA viruses rely on helicases to unwind double-stranded RNA during replication, to disassociate RNA secondary structures, such as G-quadraplexes, and to remove other RNA-bound proteins that could halt replication.^2−5^ The causative agent of the recent COVID-19 pandemic, SARS-CoV-2, is a positive sense single-stranded RNA virus that belongs to the *Betacoronavirus* genus. Within the genus, a viral helicase called nonstructural protein 13 (Nsp13) is conserved. Nsp13 helicases from the clinically relevant coronaviruses, Middle East respiratory syndrome coronavirus (MERS-CoV) and SARS-CoV-1, have 70% and 99.8% sequence conservation with SARS-CoV-2 Nsp13, respectively. Along with RNA-dependent RNA polymerase (RdRp) and additional accessory factors, Nsp13 plays an important role in the replication-transcription complex (RTC) that promotes the amplification of viral particles via RNA replication (Figure 1A).^6^ Previous reports have shown that a small molecule inhibitor (SSYA10-001) of SARS-CoV-1 Nsp13 is able to block viral replication in host cells,^7^ and is also effective against MERS-CoV and a murine coronavirus called mouse hepatitis virus.^8^ Hence, helicases are putative targets for the development of broadly effective treatments for coronavirus-related illnesses.^9^

Nsp13 is a Superfamily 1B (SF1B) helicase that is composed of five domains (Figure 1B). These domains consist of a zinc binding domain, which coordinates three Zn^2+^ ions, as well as the structural stalk domain that is connected through the 1B domain to the 1A and 2A RecA-like core helicase subdomains.^10^ The nucleotide binding site is formed by a cleft between the 1A and 2A subdomains. In vitro experiments demonstrate that one of the Zn^2+^ binding sites may instead ligate a [Fe_4_S_4_] cluster, which helps mediate RNA binding and causes sensitivity to nitroxide reagents.^11^ While there are several differences in proposed models for ATP-catalyzed unwinding of RNA by Nsp13, all suggest that one nucleotide is hydrolyzed per single base pair unwound.^10,12,13^ Similar to other ATP-powered proteins, structure-based analysis indicates that ATP hydrolysis in the 1A/2A cleft mediates the formation of open/closed conformational states in the RecA-like domains that drive translocation.^10^ Perturbation of allosteric communication between these domains represents a potential mechanism to disable viral replication in cells, although this mode of inhibition has not yet been characterized.

Several putative small molecule inhibitors of SARS-CoV-2 Nsp13 have been reported recently. Notably, a fragment-based crystallographic screen identified 65 small ligands for Nsp13, with the nucleotide binding cleft and RNA interface identified as hot spots for interactions, along with a potential allosteric binding site located between the zinc finger and stalk domains.^10^ However, none of these compounds were validated as inhibitors of helicase or ATPase activity. While diketo acids,^14^ some natural products,^15,16^ and repurposed drugs^17^ were shown to inhibit Nsp13 activity and/or SARS-CoV-2 replication in infection models, we still have a limited arsenal of reagents known to bind directly to coronavirus helicases, which might also be used to probe function and allostery. Furthermore, there are still no FDA-approved antivirals that target helicase function.^18−21^ Hence, the discovery of additional inhibitors, or activators, of Nsp13 would provide much needed small molecule tools to regulate helicase function in vitro and in cells.

Here, we develop computational approaches to identify ligand binding sites in Nsp13, and virtually screen compound libraries to discover novel inhibitors. One of the identified compounds, a clinically used drug called IOWH-032, is found to inhibit Nsp13 dual ATPase and helicase activities at micromolar concentrations. While IOWH-032 has recently been proposed to inhibit SARS-CoV-2 replication by an undefined mechanism in bronchial epithelial cells,^22^ our work suggests that Nsp13 is a possible alternative target of this compound.

## Results and Discussion

### A Computational Screen Leads to the Discovery of a Small Molecule Helicase Inhibitor

We sought to use a structure-based virtual screening campaign to identify small molecule ligands of Nsp13. At the time, the solved structure of only SARS-CoV-1 Nsp13 (PDB ID: 6JYT) was available.^23^ We reasoned this structure could be used as a screening target since SARS-CoV-1 and SARS-CoV-2 Nsp13 share 99.8% sequence identity with only one amino acid difference (I570 V), which is located distal from the protein’s active sites.^24^ ATP- and RNA-binding surfaces that could serve as potential ligand binding clefts were identified using AlphaSpace 2.0 (Figure 1B).^25^ We initially evaluated ∼6000 compounds from the Drug Repurposing Hub (Broad Institute) with the hope of identifying an approved drug as a possible COVID-19 treatment via helicase inhibition (Figure 1C and Figure S1).^26^ Each compound was prepared as diverse and low-energy conformers that were docked to the ATP-binding site with our previously described scoring function (Lin_F9).^27,28^ We initially chose to target the ATP-binding site because we hypothesized that inhibitors of nucleotide hydrolysis would also block the helicase activity of Nsp13, and ATPase assays were readily available to evaluate potential inhibitors.

**Figure 2:**
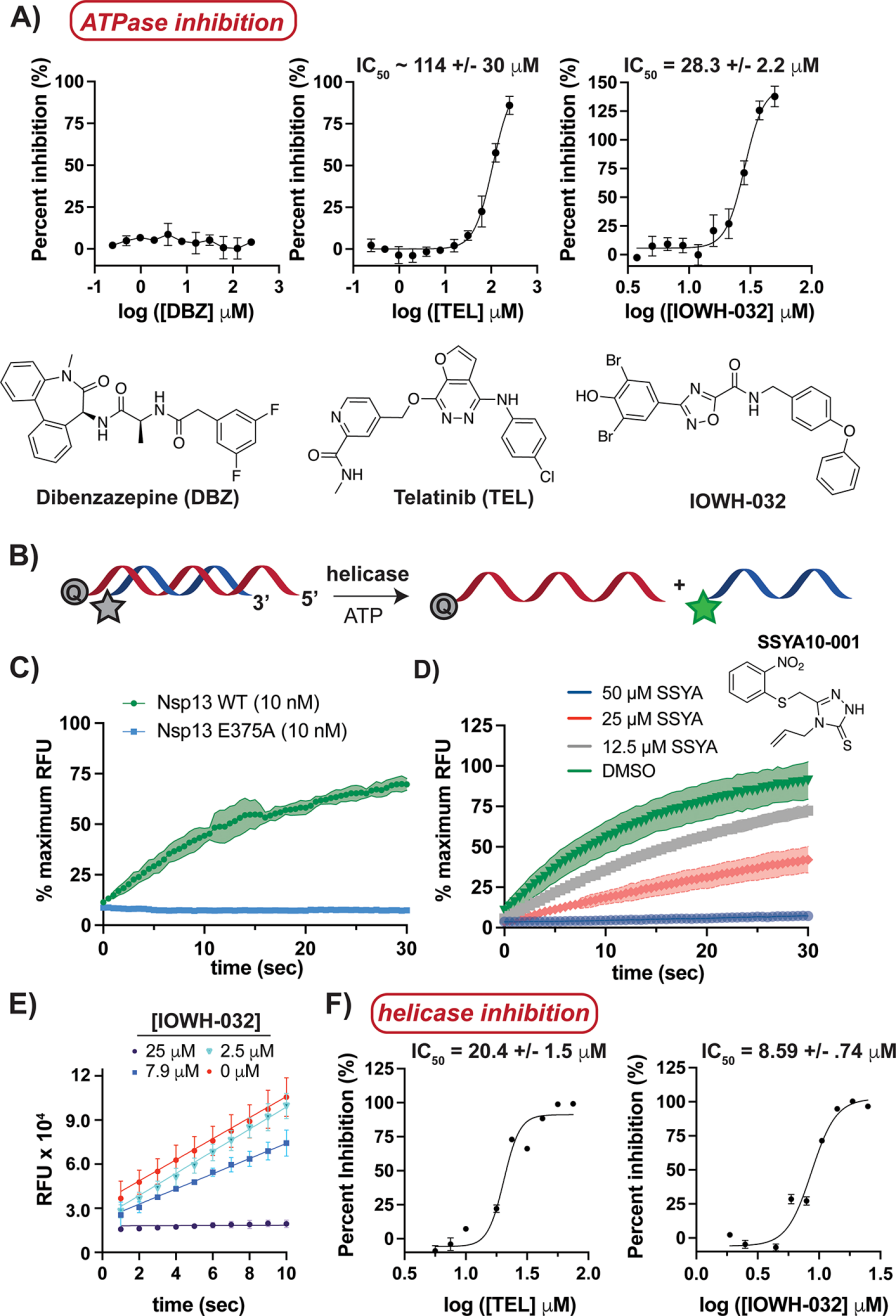
IOWH-032 inhibits Nsp13’s dual ATPase and helicase activities. (A) Titration of Nsp13 (25 nM) with indicated hits from the virtual screen demonstrates that IOWH-032 is the most potent inhibitor of ATPase activity in the presence of detergent (0.01% Tween-20). (B) Schematic of FRET-based helicase assay used, in which activity is indicated by an increase in fluorescence of labeled DNA (Q represents quencher, green star represents a functional fluorophore). (C) Analysis of helicase activity demonstrates that Nsp13 wild-type (WT) can unwind DNA in the presence of ATP, while a catalytic point mutant (E375A) does not. (D, E) Titration assays indicate that control compound (SSYA10-001) (25 nM Nsp13) and newly identified IOWH-032 (3.12 nM Nsp13) inhibit helicase activity in a concentration-dependent manner. (F) Titration of indicated compounds into Nsp13 (3.12 nM) helicase reactions with excess ATP indicates that IOWH-032 is a low micromolar inhibitor in the presence of detergent (0.01% Tween-20), demonstrating >2-fold potency over TEL. Bars and shaded regions indicate standard deviation (S.D.) for *n* = 3 replicates; the 95% confidence interval for IC_50_ values is shown. RFU = relative fluorescence units.

Following the initial screen, we experimentally evaluated 9 of the top 24 scoring compounds, which were selected based on their commercial availability (Table S4). Since compounds were docked to the ATP-binding pocket, we used luminescence-based coupled assays to assess the ability of each to inhibit the ATPase activity of purified SARS-CoV-2 Nsp13 at concentrations equimolar to that of added ATP (Figure S2). The resulting active compound structures were used to rescore the library with a machine learning scoring function, Δ_Lin_F9_XGB, for more accurate predictions (Figure S3).^29^ Out of 104 compounds that surpassed the thresholds of 4 different metrics, 21 additional commercially available compounds were tested for inhibitory activity against Nsp13 (Table S5, Figure S4). Out of the 30 total examined compounds, 14 showed partial to complete inhibition at concentrations ≤250 μM (Figures S2 and S4). Three of the most potent compounds, telatinib (TEL), IOWH-032, and dibenzazepine (DBZ), were then titrated into Nsp13 ATPase reactions in the presence of detergent (Tween-20) to reduce compound aggregation. Telatinib and IOWH-032 showed concentration-dependent ATP hydrolysis disruption with half-maximal inhibitory concentration (IC_50_) values of ∼114 and 28.3 μM, respectively (Figure 2A). However, DBZ showed no inhibition at even high micromolar concentrations in the presence of detergent, suggesting that its initial effect was due to compound aggregation.

We next examined the effect of TEL and IOWH-032 on the helicase activity of Nsp13. Although RNA is the natural substrate of Nsp13, it has been shown that DNA is an ample substitute, which also demonstrates increased stability against hydrolysis with respect to RNA.^30^ To monitor Nsp13’s 5′→3′ unwinding activity, a Cy3/BHQ2 (Cyanine 3/Black Hole Quencher 2) fluorescence resonance energy transfer (FRET)-based assay was implemented (Figure 2B). Accordingly, one DNA strand was conjugated with a Cy3 fluorescent label on the 5′ end and was annealed to a complementary strand that created a 5′ overhang and contained a 3′ BHQ2 quencher; hence, nucleic acid unwinding could be tracked by an increase in fluorescence. As expected, helicase activity was concentration-dependent and ATP-dependent (Figure S5A,B), and mutation of the ATP-binding pocket (E375A) resulted in a loss of unwinding activity (Figure 2C).^23^ The published SARS-CoV Nsp13 helicase activity inhibitor, SSYA10-001, also inhibited SARS-CoV-2 Nsp13 (Figure 2D), which further validated the experimental setup.^17,31−33^ IOWH-032 and TEL were titrated into duplex-DNA reactions initiated with excess ATP with and without detergent to assess the possible effects of compound aggregation (Figures 2E,F and Figures S5C,D). Both compounds disrupted Nsp13 helicase activity at micromolar concentrations regardless of the presence of detergent, which indicated that inhibition resulted from each compound binding to enzyme, and not aggregate formation. IOWH-032 showed >2-fold more potent inhibition of duplex DNA unwinding than TEL, and so we focused on the characterization of IOWH-032 interactions with Nsp13.

### Analogs of IOWH-032 Do Not Show Comparable Helicase Inhibition

**Figure 3:**
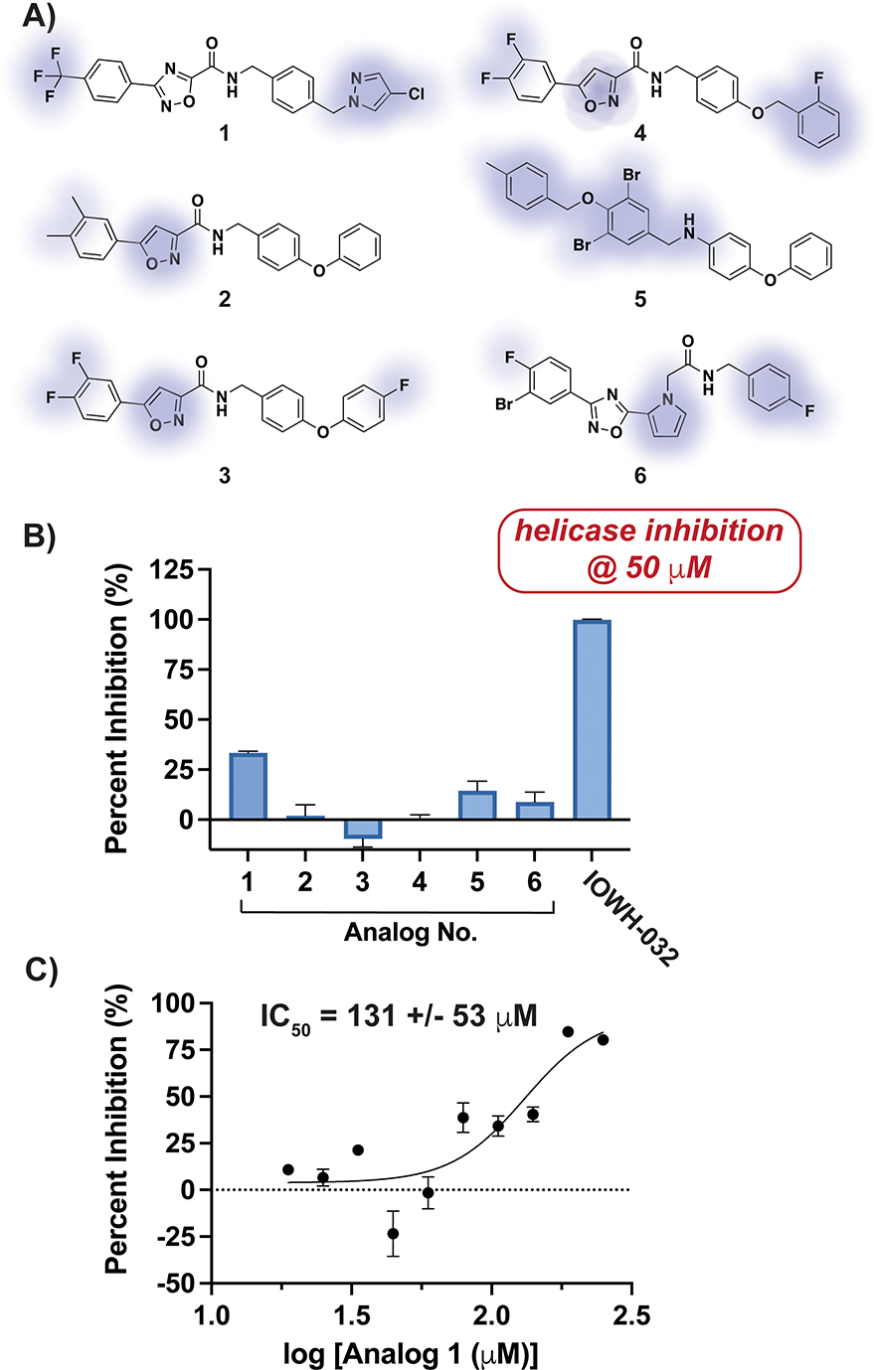
IOWH-032 structural analogs are poor inhibitors of Nsp13 helicase activity. (A) Structures of analogs selected for comparison with key differences from IOWH-032 highlighted in blue. (B) Analysis of helicase inhibition of Nsp13 (3.12 nM) by compounds in part A. (C) Helicase activity analysis indicates that compound **1** is a >10-fold less potent inhibitor than IOWH-032 of Nsp13 (3.12 nM). Tween-20 (0.01%) was added to reactions in parts B and C. Bars indicate SD (*n* = 3); the 95% confidence interval for the IC_50_ value is shown.

IOWH-032 was originally developed as a cystic fibrosis transmembrane conductance regulator (CFTR) inhibitor. The compound reached phase 2 clinical trials for the treatment of infectious diarrhea resulting from continuous activation of CFTR caused by *Vibrio cholerae* infection.^34,35^ As several structural analogs of IOWH-032 have been synthesized, we performed a focused structure–activity relationship (SAR) study with compounds containing functional group variations in different positions around the parent scaffold (Figure 3A). The tested analogs were also selected based on favorable molecular docking scores with Nsp13. Analysis of 50 μM of each analog against Nsp13 using FRET-based helicase assays indicated that the parent compound is the most potent inhibitor, and suggested that little structural variation is tolerated (Figure 3B). In particular, IOWH-032 and **2** differ only slightly in the heterocycle structure, and the nature of substituents on the phenyl ring; yet **2** shows no inhibition of Nsp13 at mid-micromolar concentration. Analogs **5** and **6**, which show the greatest structural variation from IOWH-032, showed some inhibition (<25%), but this may have resulted from a different mode of binding. Only compound **1** demonstrated >25% inhibition when added to helicase reactions, but with an IC_50_ > 10-fold higher than that of IOWH-032 (Figure 3C). Notably, **1** was the only tested compound to contain the core pharmacophore present in IOWH-032, highlighting its importance in engaging the target. Hence, we continued with the parent IOWH-032 scaffold to identify its mode of Nsp13 inhibition.

### IOWH-032 Interaction with Nsp13 Causes Displacement of Bound Oligonucleotide Substrate

**Figure 4:**
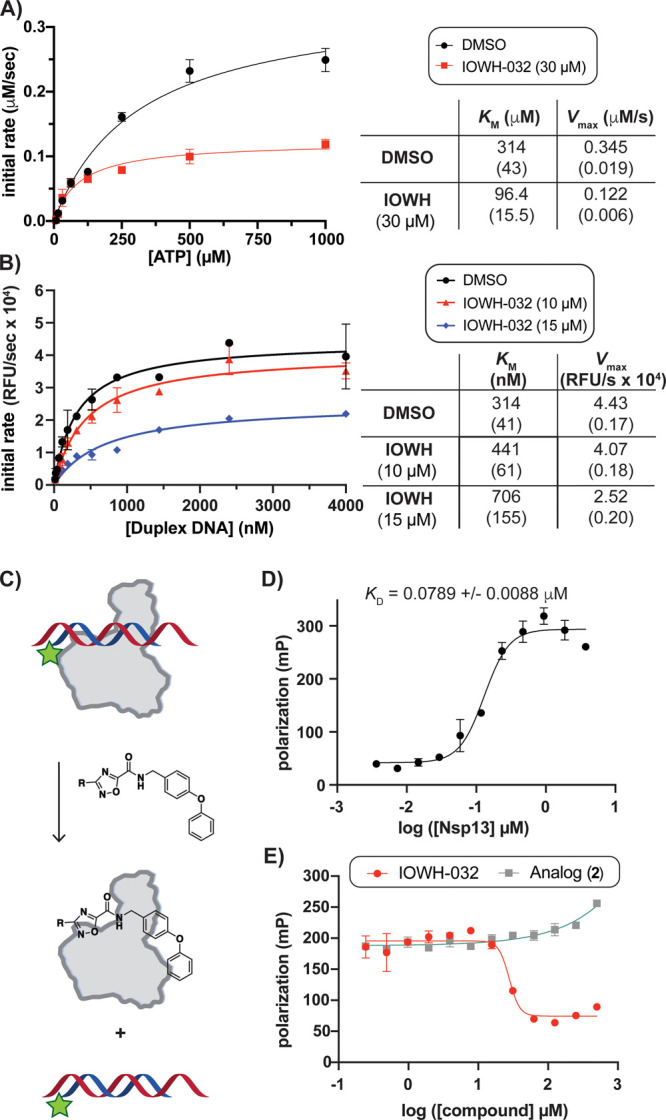
IOWH-032 interacts directly with Nsp13, leading to displacement of bound nucleic acid. (A) Michaelis–Menten analysis of Nsp13 (12.5 nM) ATPase activity with increasing [ATP] indicates an uncompetitive mode of inhibition by IOWH-032. (B) Michaelis–Menten analysis of Nsp13 (25 nM) helicase activity with increasing [oligonucleotide] indicates mixed inhibition by IOWH-032. (A,B: Standard error is shown in parentheses.) (C) Schematic of fluorescence polarization (FP)-based assay to evaluate displacement of labeled duplex DNA (duplex-FL) from Nsp13. (D) Titration of Nsp13 into solution containing 100 nM duplex-FL. (E) FP displacement assay with increasing [compound] indicates that IOWH-032 causes release of duplex-FL (100 nM) from Nsp13 (1 μM), while **2** does not. Bars and ± value indicate SD, except in part B where they represent standard error of the mean (*n* = 3 for all, except *n* = 2 for parts B and D).

In the computational small molecule screen, IOWH-032 was predicted to bind directly to the ATP binding pocket; thus, kinetic assays were initially performed to assess if IOWH-032 was competitive with ATP. Michaelis–Menten analysis was carried out after titrating ATP into Nsp13 reactions in the absence and presence of 30 μM of IOWH-032 (Figure 4A), which is near the IC_50_ value for ATPase inhibition (Figure 2A). We determined *K*_M_ and *k*_cat_ values (314 μM and 27.6 s^–1^) that were higher than those previously reported (68 μM and 0.31 s^–1^, respectively),^36^ as different protein constructs and analytical techniques were used. Furthermore, a range of *K*_M_ values for ATP from high nanomolar to low micromolar have also been published for Nsp13.^15,17,37,38^ Upon addition of IOWH-032, both the *K*_M_ and *V*_max_ values decreased by a similar factor compared to those of vehicle only reactions (3.3-fold and 2.8-fold, respectively), suggesting an uncompetitive mode of ATPase inhibition. Analysis of another submaximal concentration of IOWH-032 with Nsp13 reinforced this observation (Figure S6A). These data suggested that IOWH-032 allosterically stalled ATP hydrolysis by binding to a remote site of Nsp13 in the ATP-bound state, but did not compete with ATP as originally hypothesized.

To assess if IOWH-032 interacts with the nucleic acid binding site, we next measured Nsp13 kinetics while titrating double-stranded DNA substrate (Cy3/BHQ2-labeled) in the absence and presence of mid-micromolar concentrations of compound. We determined a *K*_M_ of ∼314 nM with only vehicle added. Notably, others have also reported nanomolar *K*_M_ values using double-stranded DNA substrate.^12,17,37,39^ The *K*_M_ for nucleic acid substrate increased with increasing concentrations of IOWH-032, while the *V*_max_ decreased, suggesting a mixed inhibition mechanism with nucleic acid substrate (Figure 4B). Additional experiments support this mode of inhibition (Figure S6B), which suggests that IOWH-032 can bind both the free and substrate-bound enzyme, potentially at the helicase active site. Since this mechanism differed from our initial postulation, we pursued additional assays to evaluate if IOWH-032 affects nucleic acid substrate binding.

To evaluate if IOWH-032 could directly displace double-stranded oligonucleotide, we opted to enlist a fluorescence polarization (FP)-based displacement assay using labeled DNA duplex (Figure 4C). The same DNA oligonucleotide duplex sequence was utilized as in helicase assays (Figure 2B), except the duplex lacked the black-hole quencher and contained a fluorescein dye (duplex-FL). Upon titration of duplex-FL into Nsp13 alone, a binding event occurred, which resulted in an approximate *K*_D_ of 79 nM (Figure 4D). As a proof of concept, we confirmed that a scrambled double-stranded oligonucleotide sequence displaced subsaturating amounts of duplex-FL probe, as nucleic acid recognition by helicases is not sequence-specific (Figure S7A). Titration with IOWH-032 also led to displacement of duplex-FL, with a *K*_I_ ∼ 3.88 μM (Figure 4E, red), similar to the calculated IC_50_ value against Nsp13 helicase activity (Figure 2F). In comparison, titration of the inactive IOWH analog, compound **2**, into the Nsp13/duplex-FL complex showed no displacement of probe (Figure 4E, gray). Similarly, titration of IOWH-032 into solution containing duplex-FL probe without protein present did not alter the polarization (Figure S7B), demonstrating that there was no direct interaction between IOWH-032 and duplex DNA. Taken together, these observations suggested that IOWH-032 can bind to the Nsp13-ATP state, and can displace nucleic acid by binding directly at the RNA interface or to a remote site that causes release of bound oligonucleotide.

### Blind Ensemble Docking and Molecular Dynamic (MD) Simulations Identify Three Possible Binding Sites for IOWH-032

As our kinetic and displacement experiments suggested that IOWH-032 did not interact directly with the ATP-binding site, as originally predicted, we sought to identify other possible small molecule binding clefts in Nsp13. Over the past several years, many additional X-ray crystallography and cryo-electron microscopy (cryo-EM) structures of Nsp13 have been solved.^40^ To better understand the major Nsp13 structural states and to improve our predictions for ligand binding, we next analyzed the current 60 X-ray and 12 cryo-EM structures of SARS-CoV-2 Nsp13. Nsp13 monomers from cryo-EM structures of the SARS-CoV-2 RTC notably revealed three distinct conformations of Nsp13 (Figure S8, Table S6). These cryo-EM structures highlighted a broad range of Nsp13 conformational diversity, in contrast to the homogeneity of deposited X-ray structures, including the SARS-CoV-1 Nsp13 structure used for our preliminary small molecule virtual screen.

**Figure 5:**
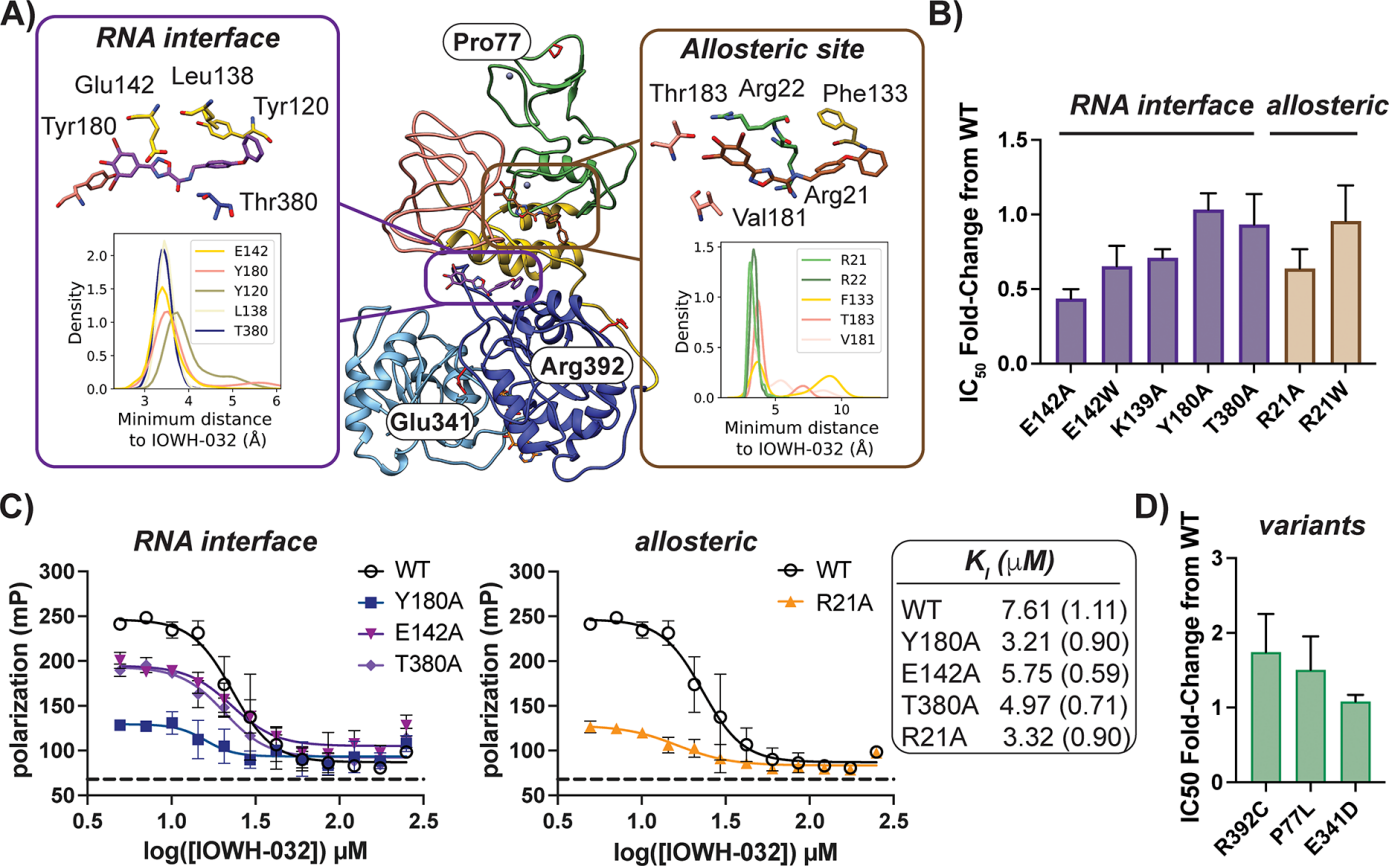
IOWH-032 interacts with the RNA interface of Nsp13. (A) Initial docked poses of IOWH-032 in the RNA (purple) and allosteric (brown) binding sites are overlaid onto Nsp13 bound to ATP (orange stick, bottom) (PDB ID: 7RDX, ATP added). Insets indicate the result of MD simulations (*n* = 6), and one frame from a single trajectory in each site is shown. A kernel density estimation was used to determine the indicated probability density distribution of the shortest distance between select side chains and IOWH-032. Note that IOWH-032 poses in the RNA and allosteric binding sites result from docking to different structures (PDB IDs: 6ZSL and 7RDX, which vary in the conformation of the 1B domain (salmon)). (B) Analysis of fold-change of IC_50_ values of select mutants in the RNA interface and allosteric site compared to wild-type indicate Ala substitutions at each site lead to enhanced sensitivity to IOWH-032. (C) FP-based competition experiments using duplex-FL (20 nM) and Nsp13 wild-type compared to indicated mutants with increasing [IOWH-032] lends further evidence that Arg21 is in an allosteric site that affects compound binding at the RNA interface near Tyr180. Note that conditions are different from those used in Figure 4D,E. Dashed line indicates probe alone. (D) Analysis of fold-change of IC_50_ values of mutants representative of SARS-CoV-2 variants versus wild-type Nsp13 indicates that IOWH-032 is still active against clinically relevant mutants. Bars indicate SD for all but B and D, for which bars indicate standard error of the mean (*n* = 3).

To explore other possible Nsp13 binding sites of IOWH-032, we performed blind docking of IOWH-032 to the full ensemble of Nsp13 structures using our scoring function for docking, in addition to a rescoring function (Δ_Lin_F9_XGB).^29^ Three potential binding sites were identified. Two overlapped with known ligand sites (ATP and RNA), and one was predicted to be an allosteric cleft that bordered the RNA binding interface (Figure 5A, Figure S9A, Table S7). We found that IOWH-032 only docked to the allosteric cleft when a “thumb” protomer (Nsp13_T_) of the Nsp13 dimer in the “1B-open” conformation was used (PDB ID: 7RDX).^41^ In this conformation, RNA does not engage with the helicase, and the 1B domain is oriented closer to the zinc-finger domain, which creates the allosteric binding cleft. Others have suggested that this conformation facilitates translocation and RNA synthesis by RdRp,^41^ which indicates that this state is important for proper cellular function. Notably, when IOWH-032 was blindly docked to an Nsp13-nucleotide analog complex (PDB ID: 7NN0), the compound directly overlapped with the analog in a favorable pose (Figure S9B). Because kinetic experiments indicated that IOWH-032 was *not* competitive with ATP, we concluded that this pose did not reflect our experimental results.

We next performed MD simulations of IOWH-032 bound to Nsp13 to evaluate possible binding poses, and to predict which amino acids may form crucial contacts with the inhibitor. The starting MD pose of the IOWH-032–Nsp13 complex was chosen following blind docking of IOWH-032 to the ensemble of Nsp13 structures, as noted above (Table S7). Since we had observed mixed inhibition of helicase activity and displacement of bound oligonucleotide by IOWH-032, we assessed compound binding at the RNA interface^10^ and the allosteric cleft with and without ATP bound (Figure S9A). With IOWH-032 bound to Nsp13 ± ATP, we measured the per-residue side chain contact frequency (Figure 5A, inset) and performed residue interaction energy analysis (Table S8). Based on these MD results, as well as kinetic experiments that suggested an uncompetitive mode of inhibition with ATP, we selected residues for mutagenesis in the RNA and allosteric binding pockets.

Four Ala mutations were made in the RNA binding cleft (E142A, K139A, Y180A, and T380A). Only one Ala mutant could be obtained in high yield in the allosteric binding cleft (R21A). To evaluate the effect of introducing a large side chain that may mimic compound binding in each pocket,^42^ E142W and R21W mutants were also purified. Most of the mutants demonstrated similar helicase rates relative to wild-type enzyme (Figure S10A–K). However, E142W and the R21A/W mutants showed an approximately 2-fold and 3–4-fold reduction in specific activity compared to wild-type, respectively (Figure S10L–N). These data suggest that while Ala mutations can be tolerated at the RNA interface, unwinding activity is sensitive to the introduction of a bulky Trp side chain. Furthermore, the observation that Arg21 mutation altered Nsp13 activity lends support to the prediction that this residue lies in an allosteric cleft that indirectly affects helicase activity.

Each of the Nsp13 mutants was then analyzed using activity and displacement assays. Evaluation of the IC_50_ values of IOWH-032 with each mutant revealed subtle changes in inhibition compared to wild-type Nsp13 (Figure 5B). The most notable shifts occurred with E142A and R21A, which had IC_50_ values ∼2-fold lower than that of wild-type enzyme, suggesting that mutation of these sites promoted binding to IOWH-032. Subtle shifts in inhibitory concentrations for another helicase inhibitor, punicalagin, have also been reported following Nsp13 mutation in the proposed binding site.^16^ FP-based analysis was then used to evaluate DNA duplex-FL probe interactions with point mutants in each potential binding pocket. Alanine substitution of residues in the RNA binding pocket (Tyr180, Glu142, and Thr380) and allosteric pocket (Arg21) all led to increased affinity for the duplex DNA probe, suggesting improved binding to nucleic acid substrate (Figure S7C–G). Different degrees of polarization with each mutant suggest that the overall conformation of each construct complexed to duplex DNA differ. Competition assays in the presence of increasing [IOWH-032] indicated that both Y180A and R21A have a > 2-fold decrease in the *K*_I_ of the inhibitor relative to wild-type (Figure 5C). In fact, all of the tested mutants demonstrated more potent interactions with IOWH-032 versus wild-type. Since IC_50_ and *K*_I_ shifts were observed in both the RNA interface and allosteric cleft, this provides further evidence that the identified allosteric cleft modulates ligand recognition at the RNA binding site.

To complement activity and displacement assays, we ran MD simulations of IOWH-032 docked to Y180A, E142A, and E142W Nsp13 mutants in the ATP bound state. We performed Molecular Mechanics-Poisson–Boltzmann (or Generalized Born) Surface Area (MM-PB/GBSA) analysis to predict binding free energies^43−45^ and to calculate the difference in binding free energies due to the introduction of select mutations (Table S9). These computational results are consistent with experimental results, as they indicate that these mutations would not significantly disrupt binding of IOWH-032 to protein, even though the selected residues were predicted to make contacts with IOWH-032 in the wild-type simulations (Figure 5A, Table S8). Our observations may stem from the flexible nature of the RNA interface, such that point mutation of IOWH-032 contact residues can be accommodated by binding site dynamics. We also initially observed many contacts breaking in the predicted allosteric docking pose, suggesting that the compound is unstable in this site (Figure 5A, inset, Figure S11). In sum, our computational results and experimental analyses of mutants provide support that the compound interacts with the RNA interaction surface of Nsp13, which is sensitive to alterations in the allosteric cleft.

### IOWH-032 Is Active in Vitro against SARS-CoV-2 Helicase Variants but Not Other Microbial Helicases

Since late 2019, viral evolution has led to SARS-CoV-2 variants that differ in genetic sequence from the original Wuhan-Hu-1 strain.^10,46−48^ Mutations in nsp13 have not been predicted to strongly enhance the overall fitness of SARS-CoV-2 when compared to mutations in other genes;^49^ as mentioned previously, Nsp13 contains only a single amino acid mutation in SARS-CoV-2 versus that of SARS-CoV-1,^50^ and Nsp13 has a low rate of mutation compared to other SARS-CoV-2 genes.^51^ Nonetheless, mutation of Nsp13 has been observed in the major viral variants, and we sought to assess the effect of these mutations on the Nsp13 inhibitory activity of IOWH-032. We chose three representative point mutations (P77L, E341D, and R392C) that were each present in the majority of sequenced Delta, Gamma, and Omicron strains, respectively (Table S10).^10,46,48^ The latter two mutations were individually predicted to promote viral fitness^48^ of their strains. None of the mutations disabled the helicase activity of Nsp13 (Figure S10I–K). Inhibitory assays showed that IOWH-032 IC_50_ values increased only slightly (∼1.5–2 fold) for the P77L and R392C mutants compared to wild-type (Figure 5D). These data suggest that compounds that target the same ligand binding cleft as IOWH-032 should be effective against major viral variants, as these mutations are present in the zinc finger domain (P77L) and subdomain 1A (R392C and E341D) (Figure 5A), which are not vicinal to the RNA interface.

Since key Nsp13 variants remain sensitive to IOWH-032, we next evaluated IOWH-032 against two diverse microbial helicases for possible inhibitory effects. While Nsp13 belongs to SF1B, the well-studied hepatitis C viral (HCV) RNA helicase NS3 belongs to superfamily 2. Reconstitution of NS3 ATPase activity in the presence of increasing IOWH-032 showed that the helicase is nearly insensitive to mid-micromolar concentrations of compound (Figure S12A), as confirmed by comparison to an inactive NS3 mutant (Figure S12B). When the superfamily 1 *Thermoanaerobacter tengcongensis* 3′→5′ DNA helicase UvrD^52^ was evaluated in the presence of IOWH-032, UvrD still demonstrated ATPase activity (Figure S12C). Hence, IOWH-032 is not a pan microbial DNA or RNA helicase inhibitor among examined proteins, and likely would not disrupt human helicases.^53^

IOWH-032 has already been examined as a therapeutic in humans;^34,35^ hence, we last aimed to evaluate the compound in a viral infection model. We first evaluated toxicity against three commonly used cell lines, two of which were derived from monkey kidney cells and one from human lung cancer cells, with and without overexpression of the human SARS-CoV-2 receptor ACE2 and associated TMPRSS2 protease (Figure S13). At only 4 μM of IOWH-032, we observed an ∼50% reduction in cell viability across all mammalian cell lines tested, preventing further analysis in infection assays. In comparison, analog **2** did not show toxicity at these concentrations, and the vehicle alone also did not inhibit growth. Since IOWH-032 is active against Nsp13 at micromolar concentrations, further compound optimization would be required for testing in common SARS-CoV-2 infection models.

## Conclusions

In this work, a virtual screen led to the identification of a drug, IOWH-032, as a potential inhibitor of the SARS-CoV-2 helicase Nsp13. Biochemical analyses demonstrated that IOWH-032 disrupts the dual ATPase and oligonucleotide unwinding activities of Nsp13. Modeling refinement complemented by kinetic and competition analyses led to the prediction of a binding cleft within the RNA binding site of Nsp13. Mutation of the RNA binding site and nearby allosteric cleft resulted in changes in the displacement of bound double-stranded DNA probe by IOWH-032. Notably, a past fragment-based crystallographic screening approach demonstrated that a small molecule fragment also bound to a hydrophobic cleft in the RNA interface that includes Tyr180, and made polar contacts with Glu142.^10^ Hence, IOWH-032 likely binds to the same pocket, which is sensitive to mutation of the allosteric cleft that may lead to broader conformational changes. Since IOWH-032 interacts with ATP-bound Nsp13, and compound binding to the RNA interface stalls ATPase activity at a distal site, IOWH-032 may act as a tool in continuing studies on helicase allostery and conformational selection mechanisms.^54^ It should also be noted that, in the course of this work, we identified an allosteric cleft from analysis of Nsp13_T_ in the “1B-open” state from structures of coronavirus proteins that had recently been solved.^55^ These results highlight the value of analyzing different conformational states of protein targets for the discovery of unique ligand binding clefts. Future virtual screening efforts focused on this site using newly solved structures and commercially available compound libraries (e.g., ZINC or Enamine)^56,57^ might lead to the discovery of new allosteric modulators.

Over the last several years, several putative inhibitors of different coronavirus Nsp13s have been reported to function both in vitro and in cells.^9^ Most of these compounds are high-nanomolar to mid-micromolar inhibitors of helicase activity. Some natural products, including myricetin and scutellarein, are micromolar inhibitors of ATPase activity, but do not affect helicase activity at similar concentrations, which has not yet been explained mechanistically.^58^ Nonetheless, myricetin is a mid-micromolar inhibitor of SARS-CoV-2 viral replication in host cells.^17^ Other known drugs that block Nsp13 activity include the antileprosy agent clofazimine,^59^ which was additionally shown to inhibit cell fusion mediated by viral spike protein, leading to high-nanomolar inhibition of viral replication in mammalian-derived Vero cells. While clofazimine is among the most potent of tested Nsp13 inhibitors in antiviral assays, this may be a consequence of its ability to perturb multiple targets. Among the characterized SARS-CoV-2 inhibitors, few have demonstrated direct binding to Nsp13, as demonstrated by displacement of duplex DNA by IOWH-032. One exception is punicalagin, a high molecular weight natural product that binds with a *K*_D_ of ∼20 nM via predicted contacts within the nucleotide binding cleft of Nsp13.^16^ However, as noted with clofazimine, punicalagin has also been implicated in disruption of other SARS-CoV-2 targets in infection models, including the 3CLpro main protease^60,61^ and viral spike-human ACE2 receptor interactions.^62,63^

Recently, two indolyl diketo acids have been synthesized that show micromolar inhibition of both the unwinding and ATPase activity of SARS-CoV-2 Nsp13, and are not known to affect other cellular targets.^14^ These compounds show noncompetitive inhibition with respect to ATP, and are predicted to bind similarly to SARS-CoV-1/-2 Nsp13 inhibitor SSYA10-001 in a RecA-like domain allosteric pocket. Accordingly, inhibitory potencies were also unaffected by the addition of extra oligonucleotide prior to compound. Like SSYA10-001, diketo acid-based compounds are recognized as “pan” coronavirus inhibitors. Low- to sub-micromolar concentrations of diketo acids block SARS-CoV-2 viral replication, as well as that of other pathogenic coronaviruses (MERS-CoV and HCoV229E) without measurable cytotoxicity against Vero cells. These other scaffolds may represent useful in vitro and cellular probes for coronavirus helicases that function by an alternative mechanism to IOWH-032.

IOWH-032 was first reported as an inhibitor of the human CFTR chloride and bicarbonate transporter for the treatment of infections related to acute watery diarrhea (AWD).^35^ Sustained activation of CFTR by bacterial toxins contributes to AWD since regulated transporter function is important for maintaining fluid homeostasis in intestinal epithelial cells. In another well-known disease state, cystic fibrosis (CF), patients contain loss of function mutations in CFTR. Interestingly, CF patients have had significantly reduced incidences of COVID^64^ and milder courses of infection during the COVID-19 pandemic.^22^ Since CF leads to chronic lung infections due to thickening of bronchial fluids, it is counterintuitive that a respiratory infection like COVID is not more detrimental to these patients. In an attempt to explain this observation, a recent study demonstrated that SARS-CoV-2 viral loads are lower in human bronchial epithelial cells that express CFTR mutants versus the wild-type. In-line with this observation, treatment of CFTR-expressing cells with micromolar concentrations of IOWH-032 led to a significant reduction (>2-log_10_) of SARS-CoV-2 viral loads compared to those in untreated cells. IOWH-032 also showed micromolar inhibition of viral replication in bronchial epithelial cells infected with a SARS-CoV-2 Omicron variant.^65^ Notably, we also found that IOWH-032 was active against the Nsp13 mutant representative of the Omicron variant. While it was originally proposed that altered CFTR function might affect host ACE2 receptor presentation and viral entry, addition of IOWH-032 at different stages of infection suggested that the drug affects the intracellular phases of viral replication. The authors proposed that alteration of the host’s intracellular pH and ionic states through disruption of CFTR could alter the ability of SARS-CoV-2 to replicate and egress from host cells, which may represent only a partial explanation of the effects of IOWH-032 on cells infected with coronavirus.

The findings of our study suggest that IOWH-032 may also abrogate viral replication in the host through inhibition of Nsp13. While we found that IOWH-032 is toxic against some commonly used human and monkey host infection cell lines, the CFTR studies used different cell lines, consisting mainly of human bronchial cells expressing different variants of CFTR.^22,65^ Furthermore, IOWH-032 is not toxic at micromolar concentrations in Chinese hamster ovary (CHO) cells expressing CFTR or colon carcinoma cells,^35^ illustrating that the presence of CFTR and the choice of cell line are important considerations for selecting host cells for future infectivity experiments. Overall, this work sets the stage for IOWH analogs to be evaluated for their ability to alter viral helicase activity in other infection models in order to expand our arsenal of much-needed antiviral agents.

## Methods

### General Methods

Primers, plasmids, and strains used in this study are described in Tables S1–S3. Primers were purchased from Invitrogen and sequencing was performed by Genewiz. Oligonucleotide probes for helicase and fluorescence polarization assays were purchased from Integrated DNA Technologies. FPLC purification and analysis were performed using an AKTA pure 15 L instrument (GE Healthcare). For ATPase assays, ATP (ThermoFisher Scientfic) and ADP (Promega) were 99% pure and ultrapure, respectively. Data analysis and graphical generation were done in GraphPad Prism software 9.0. Graphics were made using Adobe Illustrator 2023. Protein concentrations are determined using the DC assay (Bio-Rad) with bovine serum albumin (BSA) used as a standard.

## Supporting Information Available

Detailed experimental methods; plasmid, primer, and strain lists; compound structures and inhibitor testing; summary of analyzed protein structures; computational methods, including virtual screening; mutational analyses; toxicity assays; additional FRET-based helicase assays; and FP-based displacement assays and controls (PDF)

## Abbreviations

SARS-CoV-2: severe acute respiratory syndrome coronavirus 2
DNA: deoxyribose nucleic acid
RNA: ribose nucleic acid
ATP: adenosine triphosphate
Nsp13: nonstructural protein 13
MERS-CoV: Middle East respiratory syndrome coronavirus
SARS-CoV-1: severe acute respiratory syndrome coronavirus 1
RdRp: RNA-dependent RNA polymerase
RTC: replication-transcription complex
SF1B: super family 1b
FDA: Federal Drug Administration
IC_50_: half-maximal inhibitory concentration
TEL: telatinib
DBZ: dibenzazepine
CFTR: cystic fibrosis transmembrane conductance regulator
SAR: structure–activity relationship
K_M_: Michaelis–Menten constant
*V*_max_: maximal velocity
DMSO: dimethyl sulfoxide
FP: fluorescence polarization
FL: fluorescein
K_D_: dissociation constant
K_I_: dissociation constant for the unlabeled ligand
MD: molecular dynamic
cryo-EM: cryo-electron microscopy
MM-PB: Molecular Mechanics-Poisson–Boltzmann
GBSA: Generalized Born Surface Area
ACE2: angiotensin-converting enzyme 2
TMPRSS2: transmembrane serine protease 2
HCoV229E: human coronavirus 229E
AWD: acute watery diarrhea
CF: cystic fibrosis
