# Supplementary material for: A Repurposed Drug Interferes with Nucleic Acid to Inhibit the Dual Activities of Coronavirus Nsp13
Source: ACS Chem Biol. 2024 Jul 9;19(7):1593–603. doi: 10.1021/acschembio.4c00244 (PMC11267572; doi:10.1021/acschembio.4c00244)
Supplement: Supplementary file 1 — cb4c00244_si_001.pdf [file cb4c00244_si_001.pdf]

A repurposed drug interferes with nucleic acid to inhibit the dual activities of coronavirus Nsp13

Nathan Soper,[‡1] Isabelle Yardumian,[‡1] Eric Chen,[‡1,2] Chao Yang,[1] Samantha Ciervo,[1] Aaron L. Oom,[3] Ludovic Desvignes,[3, 4] Mark J. Mulligan,[3] Yingkai Zhang,*[1,2] Tania J. Lupoli*[1]

[1] Department of Chemistry, New York University, New York, New York 10003, United States
[2] Simons Center for Computational Physical Chemistry at New York University, New York, New York 10003, United States
[3] NYU Langone Vaccine Center, Department of Medicine, New York University Grossman School of Medicine, New York, New York 10016, United States
[4] High Containment Laboratories, Office of Science and Research, NYU Langone Health, New York, New York 10016, United States
[‡] Signifies authors with equal contribution
* Signifies corresponding authors

Table S1. Primers used in this study.

No.	Oligo name	Sequence
1	pNIC-ZB_Nsp13_E375A_r	GTAGTTGGTAGCCATAGAGATCGCGTCGAAGACCAC GATG
2	pNIC-ZB_Nsp13_E375A_f	CATCGTGGTCTTCGACGCGATCTCTATGGCTACCAAC TAC
3	pNIC-ZB_Nsp13_P77L_f	CTGCAAGTCACATAAGCTGCCGATTTCATTTCC
4	pNIC-ZB_Nsp13_P77L_r	GGAAATGAAATCGGCAGCTTATGTGACTTGCAG
5	pNIC-ZB_Nsp13_R392C_f	GTTAACGCGCGTCTCTGCGCTAAGCATTATGTT
6	pNIC-ZB_Nsp13_R392C_r	AACATAATGCTTAGCGCAGAGACGCGCGTTAAC
7	pNIC-ZB_Nsp13_E341D_f	ATCCCGGCGCGGGCGCGGGTCGATTGTTTTGACAAA TTTAAAG
8	pNIC-ZB_Nsp13_E341D_r	CTTTAAATTTGTCAAAACAATCGACCCGCGCCCGCGC CGGGAT
9	pNIC-ZB_Nsp13_Y180A_f	CCGTTAAACCGCAACGCGGTGTTCACCGGCTAT
10	pNIC-ZB_Nsp13_Y180A_r	ATAGCCGGTGAACACCGCGTTGCGGTTTAACGG
11	pNIC-ZB_Nsp13_K139A_f	GCGGCCGAAACCCTGGCGGCAACCGAAGAAAC
12	pNIC-ZB_Nsp13_K139A_r	GTTTCTTCGGTTGCCGCCAGGGTTTCGGCCGC
13	pNIC-ZB_Nsp13_T380A_f	CGAAATCAGCATGGCCGCGAACTACGATCTGTC
14	pNIC-ZB_Nsp13_T380A_r	GACAGATCGTAGTTCGCGGCCATGCTGATTTCG
15	pNIC-ZB_Nsp13_E142A_f	GGCCGAAACCCTGAAAGCAACCGCGGAAACGTTCAA ACTGAGC
16	pNIC-ZB_Nsp13_E142A_r	GCTCAGTTTGAACGTTTCCGCGGTTGCTTTCAGGGTT TCGGCC
17	pNIC-ZB_Nsp13_E142W_f	GGCCGAAACCCTGAAAGCAACCTGGGAAACGTTCAA ACTGAGC
18	pNIC-ZB_Nsp13_E142W_r	GCTCAGTTTGAACGTTTCCCAGGTTGCTTTCAGGGTT TCGGCC
19	pNIC-ZB_Nsp13_R21A_f	GCCTGCATCGCGCGCCCATTTCTGTGCTGTAA
20	pNIC-ZB_Nsp13_R21A_r	TTACAGCACAGAAATGGGCGCGCGATGCAGGC
21	pNIC-ZB_Nsp13_R21W_f	CGCCTGCATCTGGCGCCCATTTCTGTGCTGTAA
22	pNIC-ZB_Nsp13_R21W_r	TTACAGCACAGAAATGGGCGCCAGATGCAGGCG
23	pNIC-ZB_Nsp13_F133A_f	GAACGTCTTAAACTCGCGGCGGCCGAAACCCTG
24	pNIC-ZB_Nsp13_F133A_r	CAGGGTTTCGGCCGCCGCGAGTTTAAGACGTTC
25	pETHisSUMO_NS3_f	GAGGCTCACAGAGAACAGATTGGTGGGTAGATTTC GTCCCCGTCGAAAG
26	pETHisSUMO_NS3_r	CTTTCGGGCTTTGTTAGCAGCCGGATCAGTCACGTAA CAACCTCAAGATCGGCAGACATACACG
27	pETHisSUMO_NS3_K210N _f	CCCACCGGTTCTGGAAACTCTACGAAAGTACCC
28	pETHisSUMO_NS3_K210N _r	GGGTACTTTCGTAGAGTTTCCAGAACCGGTGGG

29	pET28a_6xHis_PreScission_SARS-CoV-2_nsp13_E375A_f	CATCGTGGTCTTCGACGCGATCTCTATGGCTACC
30	pET28a_6xHis_PreScission_SARS-CoV-2_nsp13_E375A_r	GGTAGCCATAGAGATCGCGTCGAAGACCACGATG

Table S2. Plasmids used in this study.

No.	Plasmid name	Relevant Features	Construction
1	pNIC-ZB Covid-SARS2 Nsp13	Bacterial expression plasmid for COVID-SARS2 NSP13 helicase with His_6-Zb-TEV Tag (N-Terminal on backbone) (kan^R)	Addgene #159614[1,9]
2	pET His6 Sumo TEV LIC cloning vector (2S-T)	His6-Sumo-TEV (N terminal on backbone) with no gene insert ($carb^R$)	Addgene #29711[2]
3	pET28a 6xHis PreScission SARS-CoV-2 nsp13	N-terminal His tag PreScission SARS-CoV-2 nsp13, optimized for insect cell expression(kan^R/cam^R)	Addgene #159390[3,8]
4	pNJS476	pNIC-ZB Covid-SARS2 Nsp13 E375A (kan^R)	Mutagenesis of pNIC-ZB Covid-SARS2 Nsp13 using pNIC-ZB_Nsp13_E375A_f and pNIC-ZB_Nsp13_E375A_r
5	pSRC323	pET28a 6xHis PreScission SARS-CoV-2 nsp13 E375A (kan^R/cam^R)	Mutagenesis of pET28a 6xHis PreScission SARS-CoV-2 nsp13 using pET28a_6xHis_PreScission_SARS-CoV-2_nsp13_E375A_f and pET28a_6xHis_PreScission_SARS-CoV-2_nsp13_E375A_r
6	pIY477	pNIC-ZB Covid-SARS2 Nsp13 R392C (kan^R)	Mutagenesis of pNIC-ZB Covid-SARS2 Nsp13 using pNIC-ZB_Nsp13_R392C_f and pNIC-ZB_Nsp13_R392A_r
7	pIY480	pNIC-ZB Covid-SARS2 Nsp13 Y180A (kan^R)	Mutagenesis of pNIC-ZB Covid-SARS2 Nsp13 using pNIC-ZB_Nsp13_Y180A_f and pNIC-ZB_Nsp13_Y180A_r
8	pIY487	pNIC-ZB Covid-SARS2 Nsp13 P77L (kan^R)	Mutagenesis of pNIC-ZB Covid-SARS2 Nsp13 using pNIC-ZB_Nsp13_P77L_f and pNIC-ZB_Nsp13_P77L_r
9	pIY488	pNIC-ZB Covid-SARS2 Nsp13 K139A (kan^R)	Mutagenesis of pNIC-ZB Covid-SARS2 Nsp13 using pNIC-ZB_Nsp13_K139A_f and pNIC-ZB_Nsp13_K139A_r

10	pIY517	pNIC-ZB Covid-SARS2 Nsp13 T380A (kan^R)	Mutagenesis of pNIC-ZB Covid-SARS2 Nsp13 using pNIC-ZB_Nsp13_T380A_f and pNIC-ZB_Nsp13_T380A_r
11	pIY519	pNIC-ZB Covid-SARS2 Nsp13 R21A (kan^R)	Mutagenesis of pNIC-ZB Covid-SARS2 Nsp13 using pNIC-ZB_Nsp13_R21A_f and pNIC-ZB_Nsp13_R21A_r
12	pIY521	pNIC-ZB Covid-SARS2 Nsp13 E142W (kan^R)	Mutagenesis of pNIC-ZB Covid-SARS2 Nsp13 using pNIC-ZB_Nsp13_E142W_f and pNIC-ZB_Nsp13_E142W_r
13	pIY523	pNIC-ZB Covid-SARS2 Nsp13 R21W (kan^R)	Mutagenesis of pNIC-ZB Covid-SARS2 Nsp13 using pNIC-ZB_Nsp13_R21W_f and pNIC-ZB_Nsp13_R21W_r
14	pIY524	pNIC-ZB Covid-SARS2 Nsp13 F133A (kan^R)	Mutagenesis of pNIC-ZB Covid-SARS2 Nsp13 using pNIC-ZB_Nsp13_F133A_f and pNIC-ZB_Nsp13_F133A_r
15	pIY531	pNIC-ZB Covid-SARS2 Nsp13 E142A (kan^R)	Mutagenesis of pNIC-ZB Covid-SARS2 Nsp13 using pNIC-ZB_Nsp13_E142A_f and pNIC-ZB_Nsp13_E142A_r
16	pIY342	pNIC-ZB Covid-SARS2 Nsp13 E341D (kan^R)	Mutagenesis of pNIC-ZB Covid-SARS2 Nsp13 using pNIC-ZB_Nsp13_E341D_f and pNIC-ZB_Nsp13_E341D_r
17	pTEV	pMHTDelta238-TEV Protease (kan^R)	Gift from the Traaseth Lab at NYU
18	pHYRS52	pET-His6-*S. cerevisiae* Ulp1 (res. 403-621) (amp^R)	Addgene #31122[4, 12]
19	pIY443	pET21b HCV Full-length NS3 ($carb^R$)	Twist Biosciences
20	pIY479	pETHisSUMO HCV Truncated NS3 ($carb^R$)	Mutagenesis of pET21b HCV Full-length NS3 using pETHisSUMO_NS3_f and pETHisSUMO_NS3_r
21	pIY499	pETHisSUMO HCV Truncated NS3 K210N ($carb^R$)	Mutagenesis of pETHisSUMO HCV Truncated NS3 using pETHisSUMO_NS3_K210N_f and pETHisSUMO_NS3_K210N_r

Table S3. Strains used in this study.

No.	Strain name	Description	Source
1	BL21 (DE3)	*E. coli* competent cells for high level protein expression	Novagen
2	Rosetta (DE3)/Rosetta 2 (DE3)	*E. coli* BL21 derivative for eukaryotic codon usage	Novagen
3	Ec_pNIC-ZB_Covid-SARS2_Nsp13	*E. coli* BL21 expressing pNIC-ZB Covid-SARS2 Nsp13	Addgene #159614
4	Ec_pET_His6_Sumo_TEV_LIC_Cloning_Vector	*E. coli* Mach1 expressing His6-Sumo-TEV for cloning	Addgene #29711
5	Ec_pet28a_6xHis_PreScission_SARS-CoV-2_nsp13	*E. coli* Rosetta expressing N-terminal His tag PreScission SARS-CoV-2 nsp13	Addgene #159390
6	EcNJS476	*E. coli* BL21 expressing pNIC-ZB Covid-SARS2 Nsp13 E375A	This work
7	EcSRC323	*E. coli* Rosetta expressing pET28a 6xHis PreScission SARS-CoV-2 nsp13 E375A	This work
8	EcIY477	*E. coli* BL21 expressing pNIC-ZB Covid-SARS2 Nsp13 R392C	This work
9	EcIY480	*E. coli* BL21 expressing pNIC-ZB Covid-SARS2 Nsp13 Y180A	This work
10	EcIY487	*E. coli* BL21 expressing pNIC-ZB Covid-SARS2 Nsp13 P77L	This work
11	EcIY488	*E. coli* BL21 expressing pNIC-ZB Covid-SARS2 Nsp13 K139A	This work
12	EcIY517	*E. coli* BL21 expressing pNIC-ZB Covid-SARS2 Nsp13 T380A	This work
13	EcIY519	*E. coli* BL21 expressing pNIC-ZB Covid-SARS2 Nsp13 R21A	This work
14	EcIY521	*E. coli* BL21 expressing pNIC-ZB Covid-SARS2 Nsp13 E142W	This work
15	EcIY523	*E. coli* BL21 expressing pNIC-ZB Covid-SARS2 Nsp13 R21W	This work
16	EcIY524	*E. coli* BL21 expressing pNIC-ZB Covid-SARS2 Nsp13 F133A	This work
17	EcIY531	*E. coli* BL21 expressing pNIC-ZB Covid-SARS2 Nsp13 E142A	This work
18	Ec_pTEV	*E. coli* BL21 expressing pMHTDelta238-TEV Protease	Gift from the Traaseth Lab at NYU
19	Ec_pHYRS52	*E. coli* BL21 expressing pET-His6-*S. cerevisiae* Ulp1 (res. 403-621) (amp^R)	Addgene #31122
20	EcIY443	*E. coli* Rosetta 2 expressing pETHisSUMO HCV Truncated NS3	This work
21	EcIY479	*E. coli* Rosetta 2 expressing pETHisSUMO HCV Truncated NS3 K210N	This work

22	Vero ACE2-TMPRSS2	*Cercopithecus aethiops* kidney epithelial cells with human ACE2 and TMPRSS2 proteins overexpressed	BEI Resources NR-54970
23	Vero E6	*Cercopithecus aethiops* kidney epithelial cells	ATCC #CRL-1586
24	A549 ACE2	Human epithelial lung cell line with overexpressed ACE2 protein	de Vries et al., 2021[15]

Table S4. Structures of compounds selected for analysis from first round of screening.

Compound Name	Compound Structure	Abbreviation
Allitinib	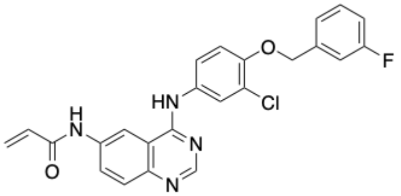	AST
Vactosertib	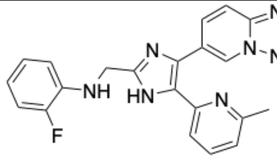	EW
LGK974	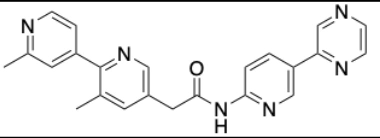	LGK
Linifanib	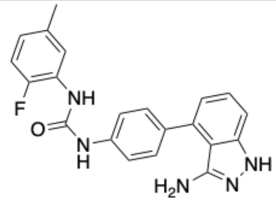	ABT
Raltegravir	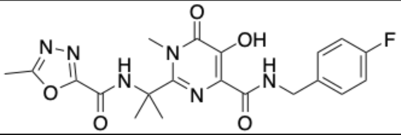	MK
Regorafenib	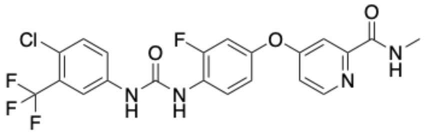	BAY
Sorafenib (tosylate)	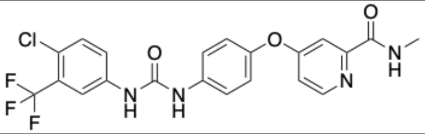	ST
Telatinib	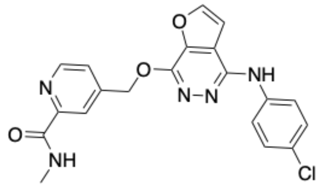	TEL
Dibenzazepine	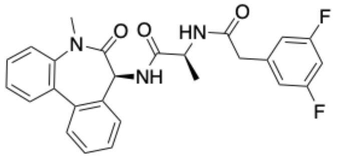	DBZ

Table S5. Structures of compounds selected for analysis from second round of screening.

Compound Name	Compound Structure	Abbreviation
AMG-517	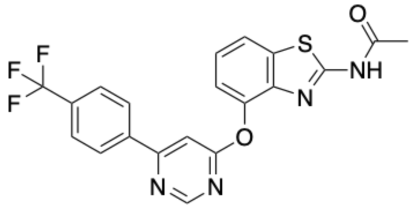	HEL-1
Varlitinib	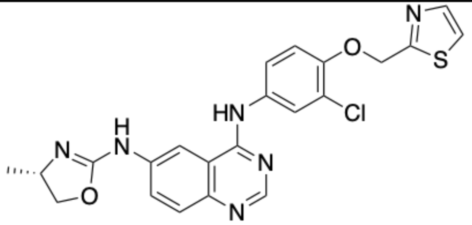	HEL-2
AVL-292	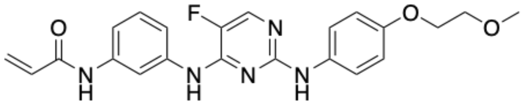	HEL-3
BMY-7378 (2HCl)	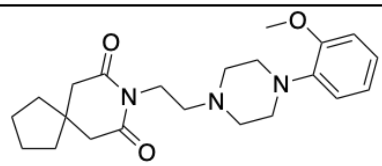	HEL-4
BX-912	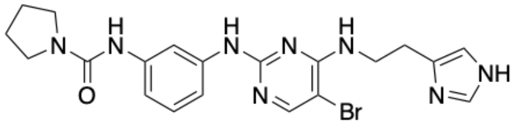	HEL-5
CCT129202	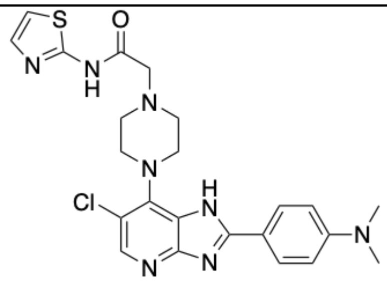	HEL-6
CNX-774	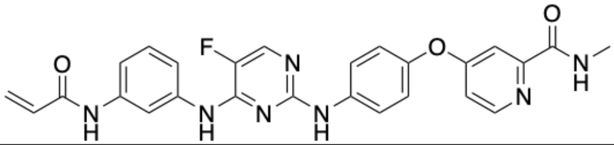	HEL-7

PF299804 (Dacomitinib)	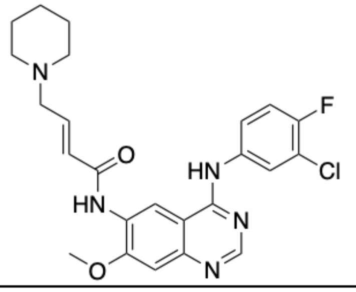	HEL-8
GSK1292263	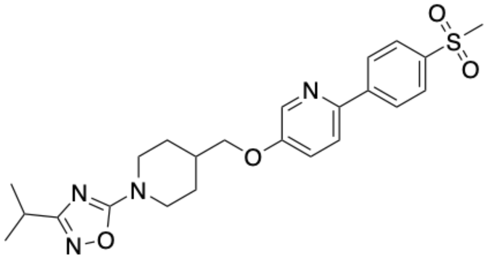	HEL-9
IOWH-032	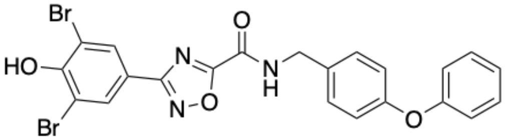	HEL-10
Fluvastatin	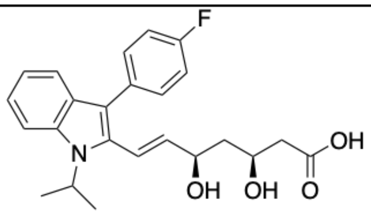	HEL-11
GNF-2	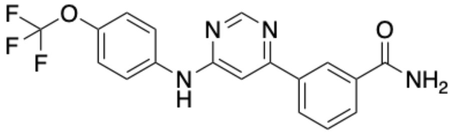	HEL-12
KPT-276	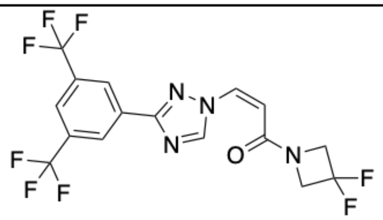	HEL-13
Lenvatinib	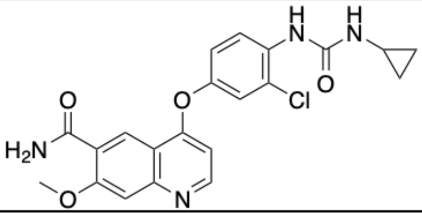	HEL-14

NVP-AEW541	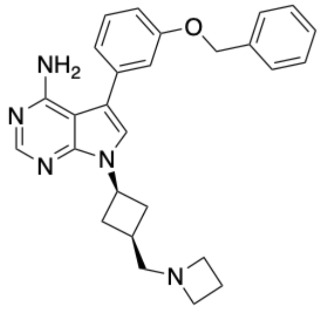	HEL-15
NVP-TAE226	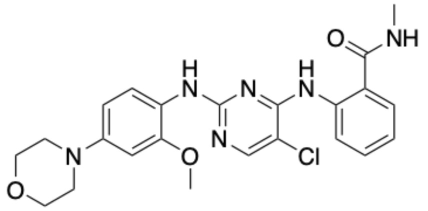	HEL-16
Omecamtiv mecarbil	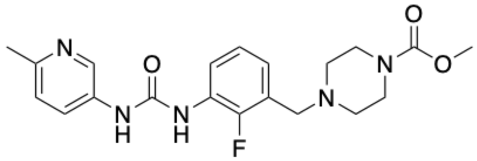	HEL-17
Roflumilast	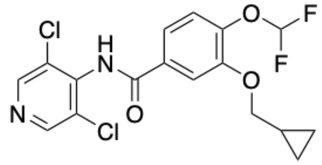	HEL-18
Tivozanib	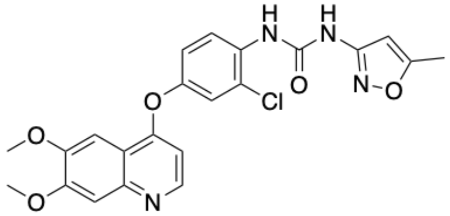	HEL-19
Vandetanib	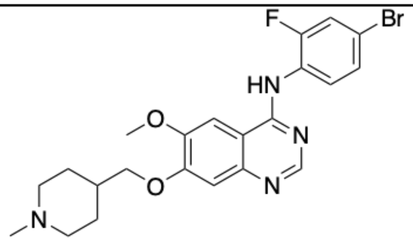	HEL-20
WZ-3146	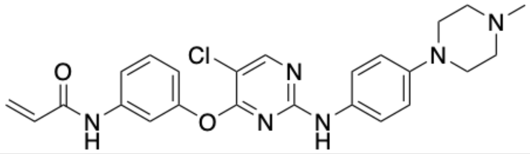	HEL-21

Table S6. PDB ID and chain of the structural ensemble of SARS-CoV-2 Nsp13 cryo-EM structures following Principal Component Analysis (PCA) and Hierarchical Density-Based Spatial Clustering of Applications with Noise (HDBSCAN).

Cluster	cryo-EM: PDB ID_chain												
C1	**7RDX_E** [b]					7RE3_E [c]	7RE3_K	*7EGQ_E* [d]	*7EGQ_R*				
C2		6XEZ_E	7RE0_E	7RE1_E	**7RDY_E**					7RE2_E	**7KRN_E**	**7KRO_E**<br>7KRO_F[a]	
C3	7RDX_F	6XEZ_F	7RE0_F	7RE1_F		7RE3_F	7RE3_L	*7EGQ_F*	*7EGQ_S*				*7RDZ_E*<br>*7RDZ_F*
Noise					7RDY_F								

[a] These structures represent the Nsp13 dimer in the "backtracked" complex
[b] Indicates chain E
[c] Structures bound by ADP are indicated in normal font
[d] Structures not bound by ADP are indicated in italics
[e] Structures which make direct contacts with RNA are bolded

Table S7. Ensemble docking pose binding affinity analysis per receptor cluster and ligand pose location cluster.

Lin_F9 + Δ_{Lin_F9}XGB		IOWH-032 Pose Binding site							
		Allosteric		RNA #1[a]		RNA #2[b]		ATP	
Cluster	# of poses [# of receptors]	*	Δ_{Lin_F9}XGB	*	Δ_{Lin_F9}XGB	*	Δ_{Lin_F9}XGB	*	Δ_{Lin_F9}XGB
C1	25 [5]	16%	6.402±0.10	16%	6.607±0.02			68%	6.520±0.12
C2	40 [8]			77%	6.574±0.21	17%	6.685±0.12	5%	6.526±0.17
C3	50 [10]			66%	6.518±0.14			34%	6.522±0.16
x-ray	305 [61]			80%	6.585±0.32	5%	6.650±0.14	13%	6.496±0.27

* Percentage of docked poses for which IOWH-032 bound to the binding site per cluster
[a] The center of the RNA binding pocket at the core of the protein
[b] The proximal region of the RNA binding site (with respect to the view seen in **Figure 1B**). This region is bordered by the 1B and 2A subdomains, and the hinge region.

Table S8. Analysis of select Nsp13 residues that contribute to IOWH-032 binding.*

Residue	ΔG_{bind}^{AA-SC}(kcal/mol) MM-PBSA	MM-GBSA	Contact Frequency
TYR 180	-0.664±0.08	-1.018±0.16	83.8±16.2
GLU 142	5.302±0.44	-0.225±0.14	99.8±0.2
LEU 138	-1.312±0.05	-1.291±0.07	100.0±0.0
THR 380	-0.91±0.05	-1.957±0.05	100.0±0.0
TYR 120	-0.56±0.09	-0.93±0.11	98.4±0.4
THR 183	0.321±0.38	-0.458±0.39	66.1±33.0
VAL 181	-0.78±0.36	-0.881±0.43	65.2±32.3
ARG 22	0.4±1.3	-1.333±0.38	99.9±0.1
ARG 21	0.2±0.61	-1.327±0.0	99.6±0.4
PHE 133	-0.668±0.26	-0.948±0.38	67.0±32.9

* Side chain contact frequency and residue interaction analysis from the WT Nsp13–IOWH-032 simulations with and without ATP bound. Average contribution of binding free energy per residue side chain ($\Delta G_{bind}^{Amino\ Acid\ (AA)-Side\ Chain\ (SC)}$) with standard error and mean of the replicates reported. This analysis assumes that removal of the side chain does not affect the structure. Purple colored text indicates results from the 5 out of the total 6 trajectories of IOWH-032 at the RNA site. Orange/brown colored text indicates 3 out of the total 6 of trajectories of IOWH-032 at the allosteric site. Removing these trajectories prioritized the determination of the residues that would interact with IOWH-032 during the analysis.

Table S9. Change in average binding free energy upon mutation of select residues ($\Delta\Delta G_{bind}^{WT\rightarrow MUT}$)*

Simulation	MM-PBSA	MM-GBSA
	ΔG^{WT} (kcal/mol)	
WT	-11.96±1.36	-39.89±0.78
Mutation	$\Delta\Delta G^{WT\rightarrow MUT}$ (kcal/mol)	
Y180A	-8.5±1.02	2.34±0.49
E142A	-15.32±1.11	-2.82±0.46
E142W	-15.62±4.21	-3.14±4.98

*Average binding free energy of IOWH-032 to the Nsp13–ATP complex (ΔG^{WT}) with standard error of the mean of the three replicates reported. Change in average binding free energy ($\Delta\Delta G_{bind}^{WT\rightarrow MUT}$) with standard errors of the difference of means. The binding free energy is calculated with Molecular Mechanics-Poisson–Boltzmann (or Generalized Born) Surface Area MM-PB/GBSA. Positive $\Delta\Delta G_{bind}^{WT\rightarrow MUT}$ suggest weaker binding and negative $\Delta\Delta G_{bind}^{WT\rightarrow MUT}$ suggest improved binding following the mutation.

Table S10. Three point mutations in Nsp13 are representative of three major SARS-CoV-2 variants.*

Mutation	Variant	Percent Prevalence within Variant
E341D	Gamma	100% (7/7 genomes)
P77L	Delta	98.9% (181/183 genomes)
R392C	Omicron	98.39% (3126/3177 genomes)

*P77L is representative of the Delta variant (clades 21I, 21J); E341D is representative of the Gamma variant (clade 20J); R392C is representative of most Omicron variants (clades 21L, 22A, 22B, 22C, 22E). Percent prevalences were calculated using the Nextstrain bioinformatics software. The 3,972-genome library was filtered by selecting the variant of choice by clade, locating the desired mutation in Nsp13 (found between the coding nucleotide location 16240-18039 in the nucleotide/amino acid diversity map) and selecting the amino acid associated with the mutation. The number of sample genomes containing that mutation were then compared to the total for that variant.[5, 6]

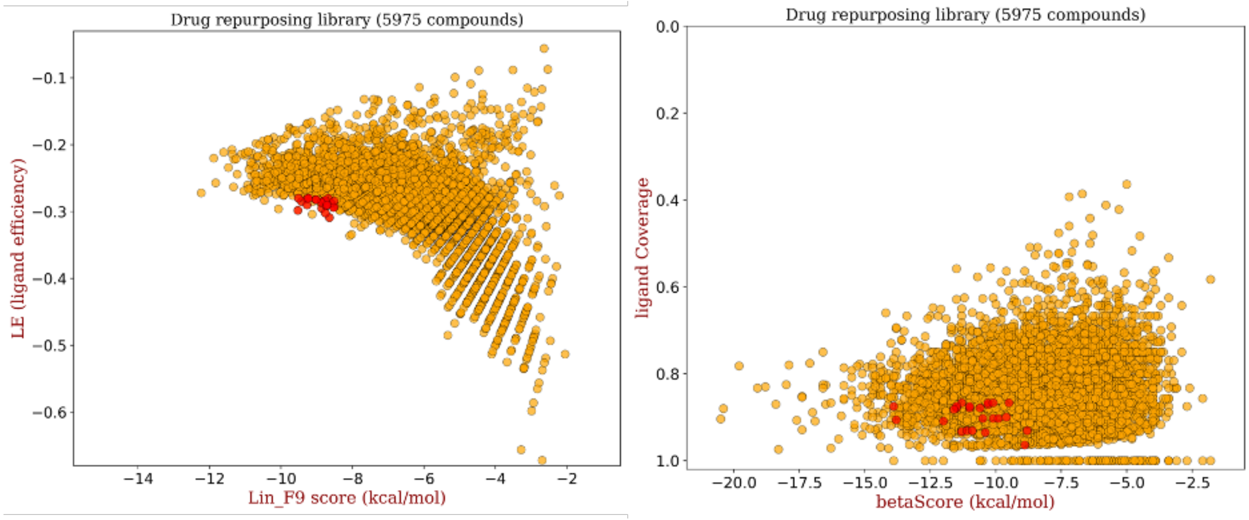

Figure S1. Virtual screening of approximately 6000 compounds from drug repurposing library using thresholds of four different metrics.[7] The left scatter plot shows the Lin_F9 score vs. ligand efficiency, while the right scatter plot shows the ligand β-score vs. ligand coverage. The compounds that had a Lin_F9 score <=-8.5 kcal/mol, ligand efficiency <= -0.28, ligand coverage >= 0.85, and ligand β-score <= -8.5 kcal/mol were selected from the screen. The selected compounds that meet the thresholds are highlighted in red.

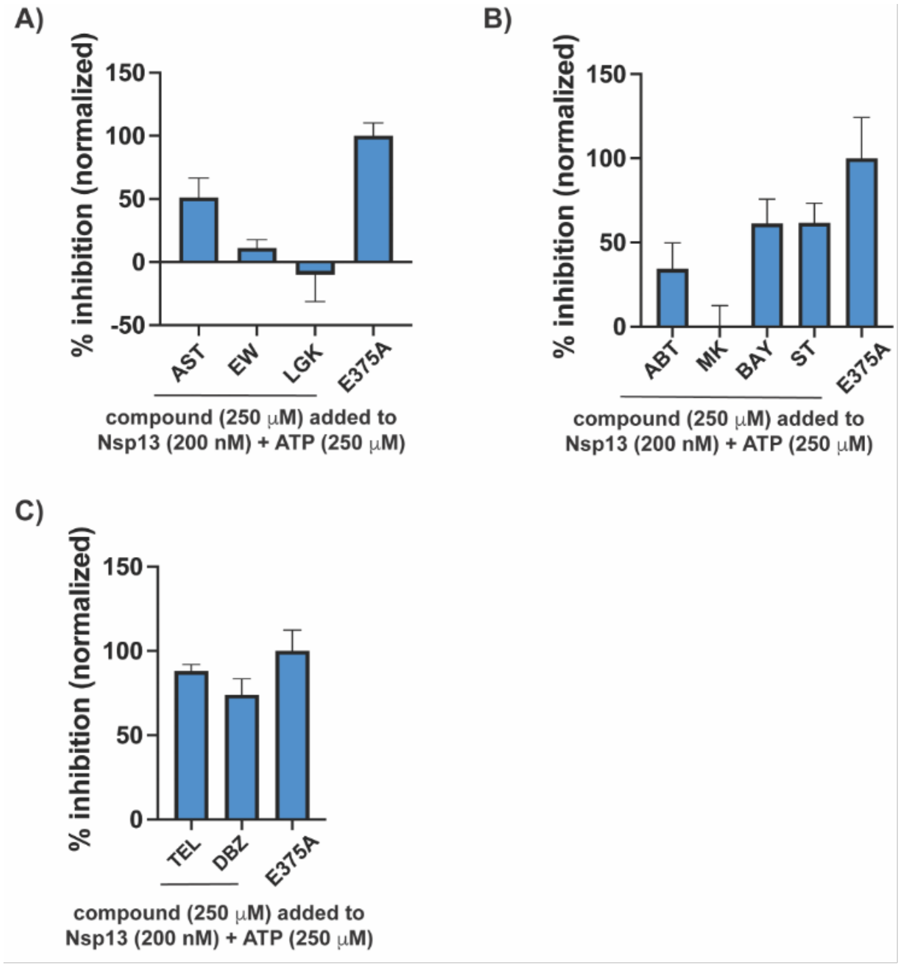

Figure S2. First round of inhibitors tested for inhibition of Nsp13 ATPase activity led to identification of initial hits. Compounds were incubated with Nsp13 (pET28a 6xHis PreScission SARS-CoV-2 Nsp13) at indicated concentrations and ATP consumption was calculated within the initial linear phase (t = 5 min). Normalized percent inhibitions were calculated based on reactions lacking compound with only vehicle (DMSO) added (0%), and reactions with inactive mutant E375A (pSRC323) containing vehicle only (100%), as shown. The following compounds were considered “active” based on this assay: AST, ABT, BAY, ST, TEL and DBZ, with the latter two showing the greatest inhibition. Structures are shown in **Table S4**. No detergent was used in these assays. Error bars represent standard deviation (SD) for n = 3 experiments.

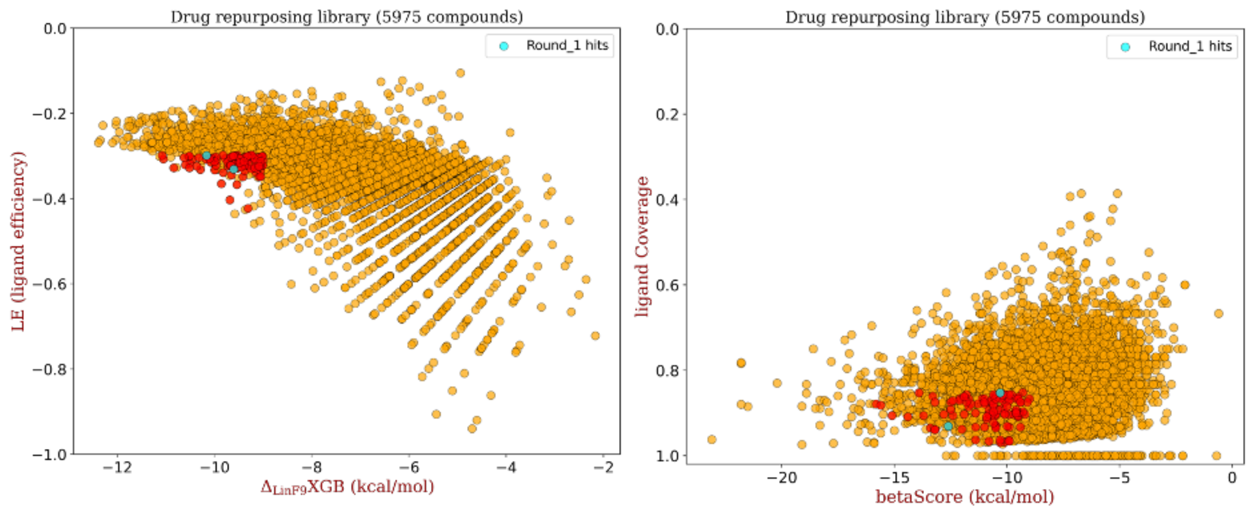

Figure S3. Virtual screening of approximately 6000 compounds from the drug repurposing library using thresholds of four different metrics and an updated scoring function. The left scatter plot shows the Δ_{Lin_F9}XGB score vs. ligand efficiency, while the right scatter plot shows the ligand β-score vs. ligand coverage. The compounds that had Δ_{Lin_F9}XGB score <= -9.0 kcal/mol, ligand efficiency <= -0.30, ligand coverage >= 0.85, and ligand β-score <= -8.5 kcal/mol were selected. The selected compounds that meet the thresholds are shown as red dots. Previously screened active compounds (telatinib (TEL) and dibenzazepine (DBZ)) are shown as cyan dots.

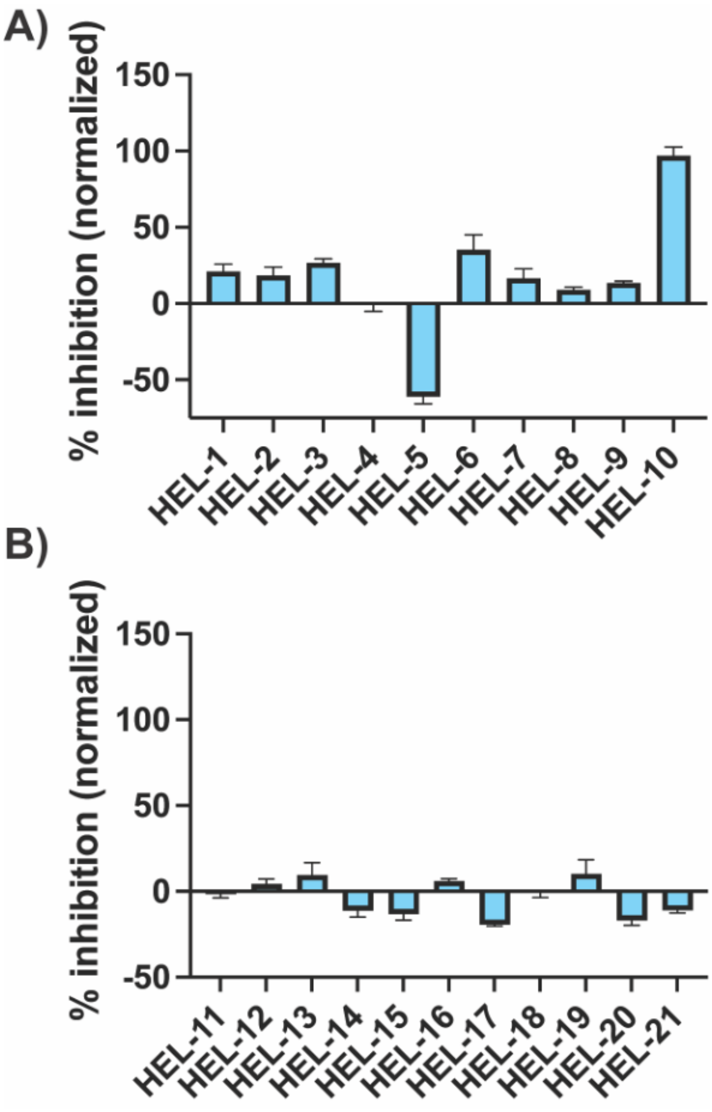

Figure S4. Second round of inhibitors tested for inhibition of Nsp13 ATPase activity leads to identification of IOWH-032. (A) Indicated compounds (100 µM) were added to reactions containing Nsp13 (pET28a 6xHis PreScission SARS-CoV-2 Nsp13) (50 nM) and 250 µM ATP, and percent inhibition of ATPase activity was measured. (B) Indicated compounds (250 µM, except for HEL-14 (200 µM) and HEL-15 (100 µM)) were added to reactions containing Nsp13 (pET28a 6xHis PreScission SARS-CoV-2 Nsp13) (25 nM) and 250 µM ATP and percent inhibition of ATPase activity was measured. 10% DMSO was present in all reactions, which were analyzed in the initial linear phase (t = 15 min). 0.01% Tween-20 was used in all reactions. Normalized percent inhibitions were calculated based on reactions lacking compound (0%) and reactions with no enzyme added (100%). Structures are shown in **Table S5**. Error bars indicate S.D. for n = 3.

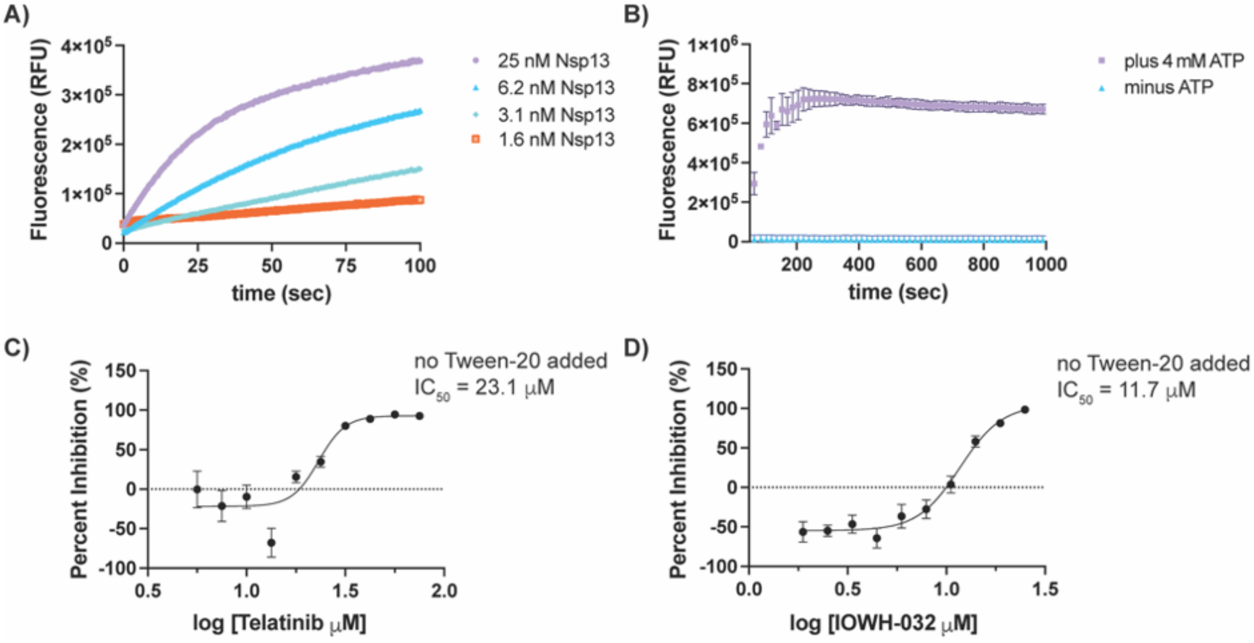

Figure S5. Helicase activity and inhibition can be measured using a FRET-based assay. (A) The rate and extent of labeled double-stranded DNA unwinding varies based on the concentration of Nsp13 used (pET28a 6xHis PreScission SARS-CoV-2 Nsp13). (B) Analysis of helicase unwinding by Nsp13 (35 nM) in the presence and absence of ATP indicates that double stranded DNA unwinding is ATP-dependent. (C-D) Titrations of Telatinib and IOWH-032, respectively, into Nsp13 helicase reactions lacking detergent. Normalization was performed by comparison to reactions lacking compound (0% inhibition, only DMSO present) and reactions lacking enzyme (100% inhibition). Experiments were performed at a final volume of 40 uL with 4 mM ATP and Nsp13 (pET28a 6xHis PreScission SARS-CoV-2 Nsp13, 3.12 nM). Error bars represent SD for n = 3 (parts B-D).

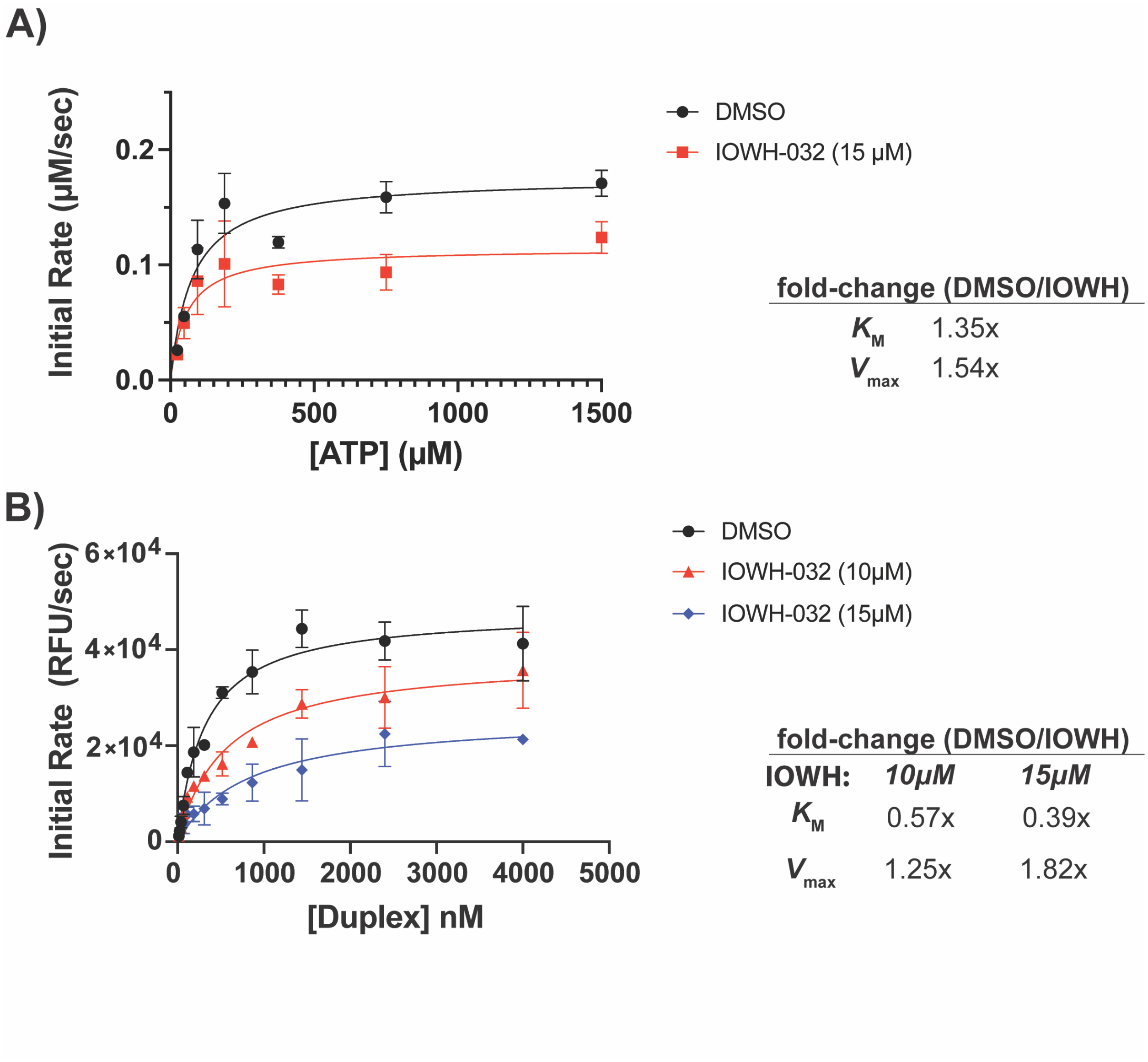

Figure S6. Kinetic analyses indicate that IOWH-032 does not compete with ATP. (A) Michaelis-Menten analysis of Nsp13 (25 nM) ATPase activity with increasing [ATP] in the presence and absence of IOWH-032 indicates an uncompetitive mode of inhibition, as seen in **Figure 4A** with a higher concentration of IOWH-032 tested. (B) Michaelis-Menten analysis of Nsp13 (25 nM) helicase activity with increasing [oligonucleotide duplex] in the presence and absence of increasing [IOWH-032] indicates mixed inhibition, as seen in **Figure 4B**. Error bars indicate SD for n = 2 in part A and standard error of the mean (SEM) for n = 2 for part B.

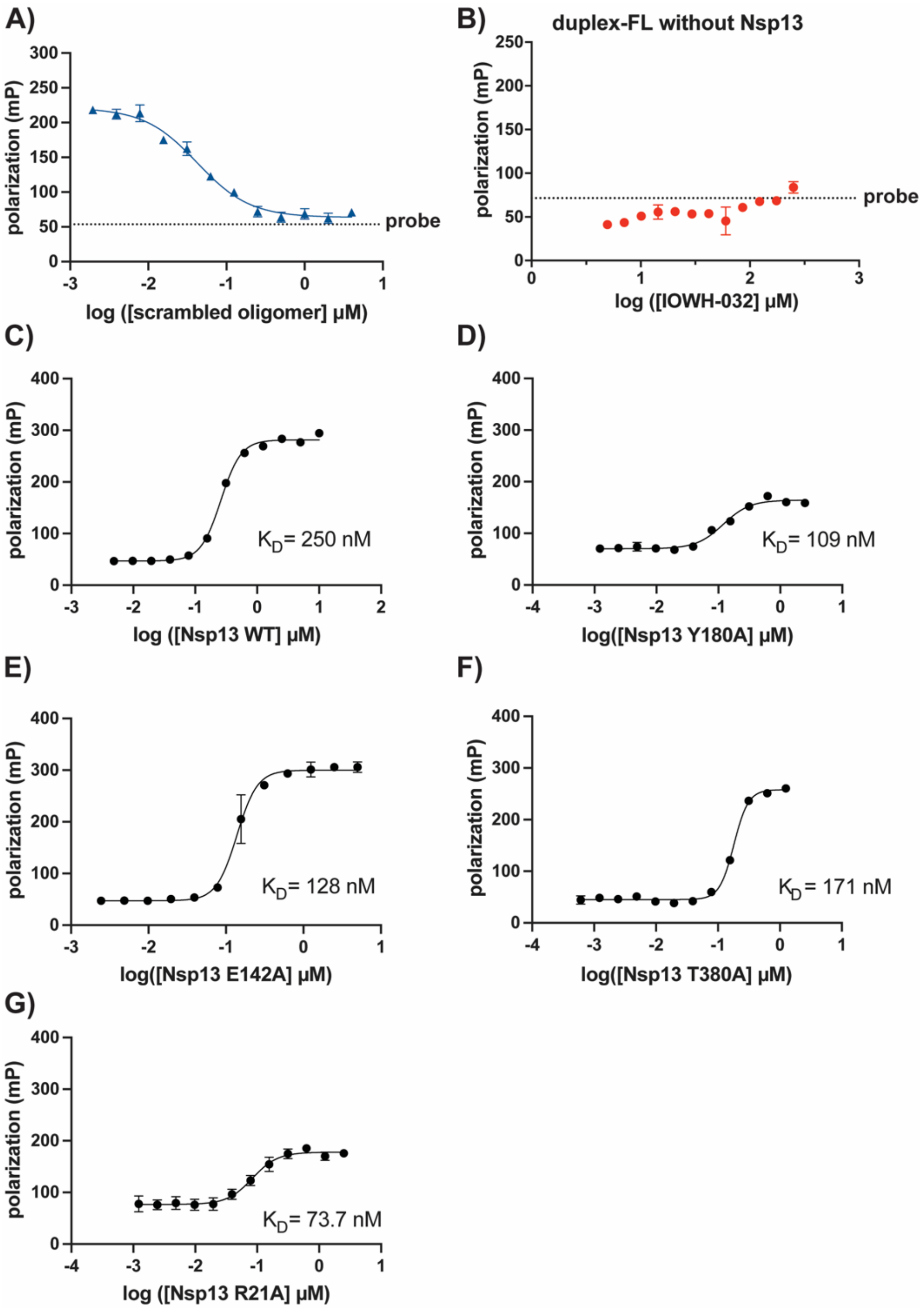

Figure S7. Fluorescence polarization (FP)-based analysis indicates that IOWH-032 competes with oligonucleotide for binding to Nsp13. (A) Competition experiment using unlabeled duplex oligonucleotide containing a scrambled sequence (based on that of the duplex DNA probe) titrated into Nsp13 (200 nM) leads to displacement of duplex-FL (100 nM) in 10% DMSO (EC_{50} = 421 nM). (B) Titration of IOWH-032 into solution containing duplex DNA-FL (100 nM) and 10% DMSO shows that IOWH-032 does not interact with probe alone. For A-B, dashed line indicates the polarization of probe only, and incubations were performed for t = 60 min each. (C-G) Saturation binding experiments for Nsp13 wild-type (WT) and indicated mutants using 20 nM of duplex-FL in 10% DMSO. Note that the calculated K_D for WT is different than that in **Figure 4D**, as a different concentration of probe was used, and incubations extended t = 30-180 min for these experiments. Error bars represent SD (n = 3 except n = 2 for part B).

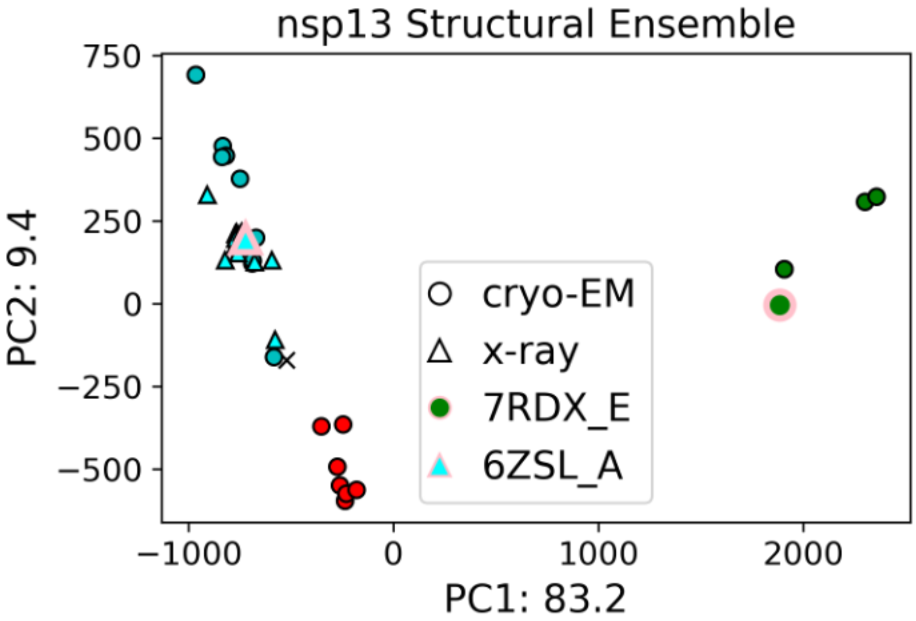

Figure S8. Structural ensemble analysis of the SARS-CoV-2 Nsp13 structures. Each structure is represented by a pairwise residue–residue shortest distance matrix. PCA and HDBSCAN are used to visualize and cluster the cryo-EM data representations. **Table S6** contains the list of the corresponding structures and chains. The X-ray crystal structures are projected onto the corresponding PCA plot. Highlighted in pink are the two structures used for the initial pose of the MD simulations. All structures are used to perform the ensemble docking of IOWH-032.

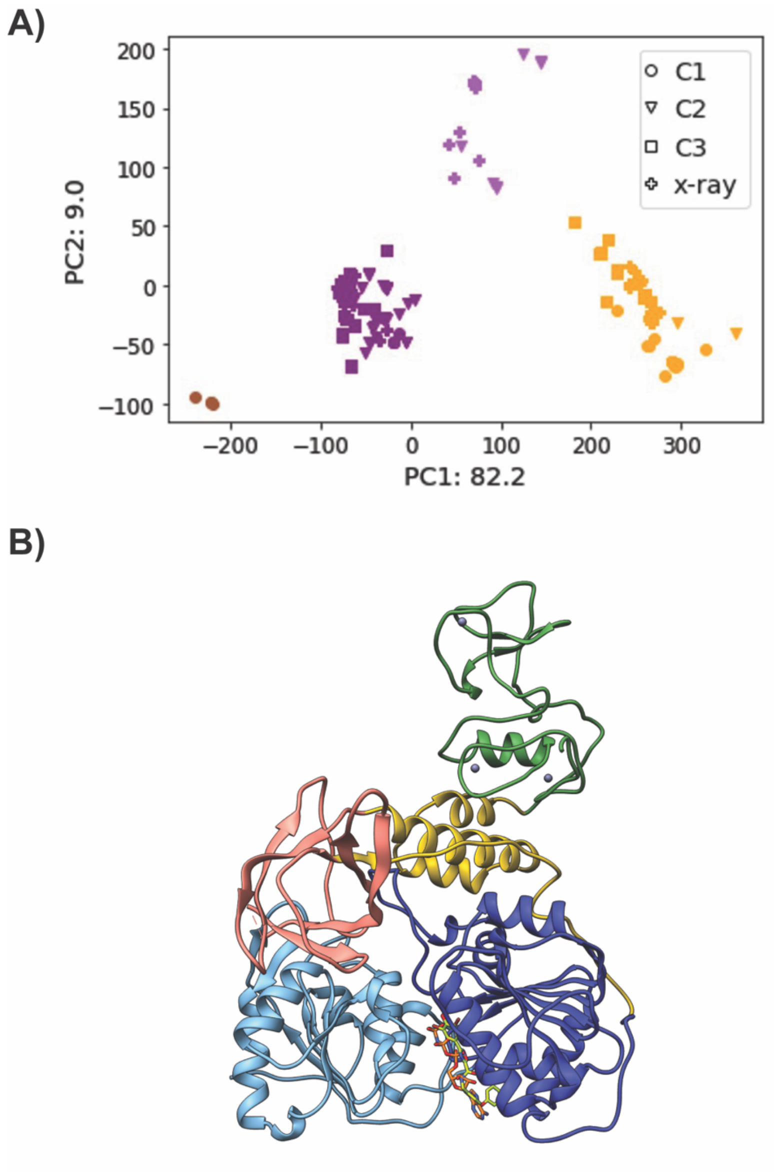

Figure S9. Ensemble docking pose analysis of the docking location of IOWH-032 to every structure of SARS-CoV-2 Nsp13 and analysis of possible ATP binding pose. (A) The top 5 poses by predicted binding affinity were selected for each receptor. Each docking pose was represented by a ligand–receptor shortest distance vector. PCA and HDBSCAN are used to visualize and cluster the docking location to three sites, allosteric (brown), RNA #1 and #2 (purple), and ATP (orange). **Table S7** contains the breakdown of the docking location, frequency, and binding affinity predictions per receptor cluster and ligand pose location cluster. (B) Top ranked pose of IOWH-032 (lime green stick) when blindly docked by Δ_{Lin_F9}XGB to an Nsp13-nucleotide analog complex (PDB ID: 7NN0) indicates that IOWH-032 directly overlaps with ANP-PNP (Adenylyl imidodiphosphate, a non-hydrolyzable ATP-analog, orange stick). This pose suggests that IOWH-032 would be competitive with ATP, which was not observed experimentally, and so other poses were pursued.

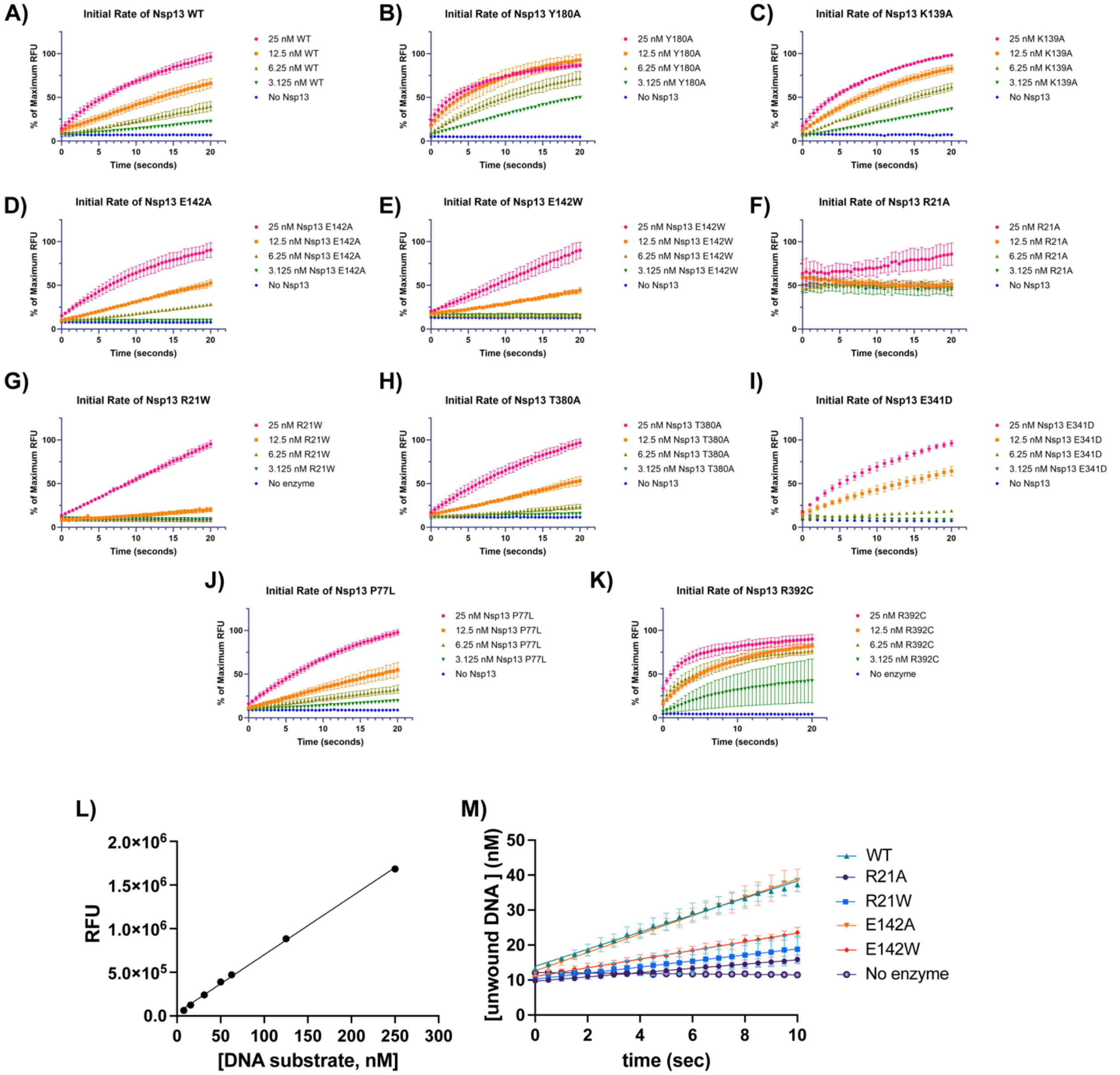

N)

protein	specific activity (nM DNA unwound/sec•nM protein)
WT	0.0975
R21A	0.0244
R21W	0.0346
E142A	0.1050
E142W	0.0496

Figure S10. Helicase mutants demonstrate helicase activity at nanomolar concentrations, some with lower rates compared to wild-type protein. (A-K) The initial rates of each tested mutant of Nsp13 at 25, 12.5, 6.25, and 3.125 nM concentrations. Each graph of was normalized based on the maximum RFU/sec value of each data set. (L) Standard curve generated by measuring the fluorescence of increasing concentrations of labeled double stranded DNA (DNA-FL: Oligomer 1-(5'-/Cy3/CCTCGTAGGTACGCATGGATCCTCT-3') and Oligomer 2-(5'-GCTTGCATGCCTGCAGGTGCACTCTAGAGGATCCATGCGTACCTACGAGG-3'). This probe mimics unquenched DNA as it unwinds via helicase activity. (M) Initial rates of indicated Nsp13

constructs at 25 nM with 50 nM DNA duplex over a 10 sec time course at 28 ºC, with the unwound DNA concentration calculated based on the curve in part A. (N) Calculated specific activities of Nsp13 constructs based on data in part L. Error bars represent SD for n = 3.

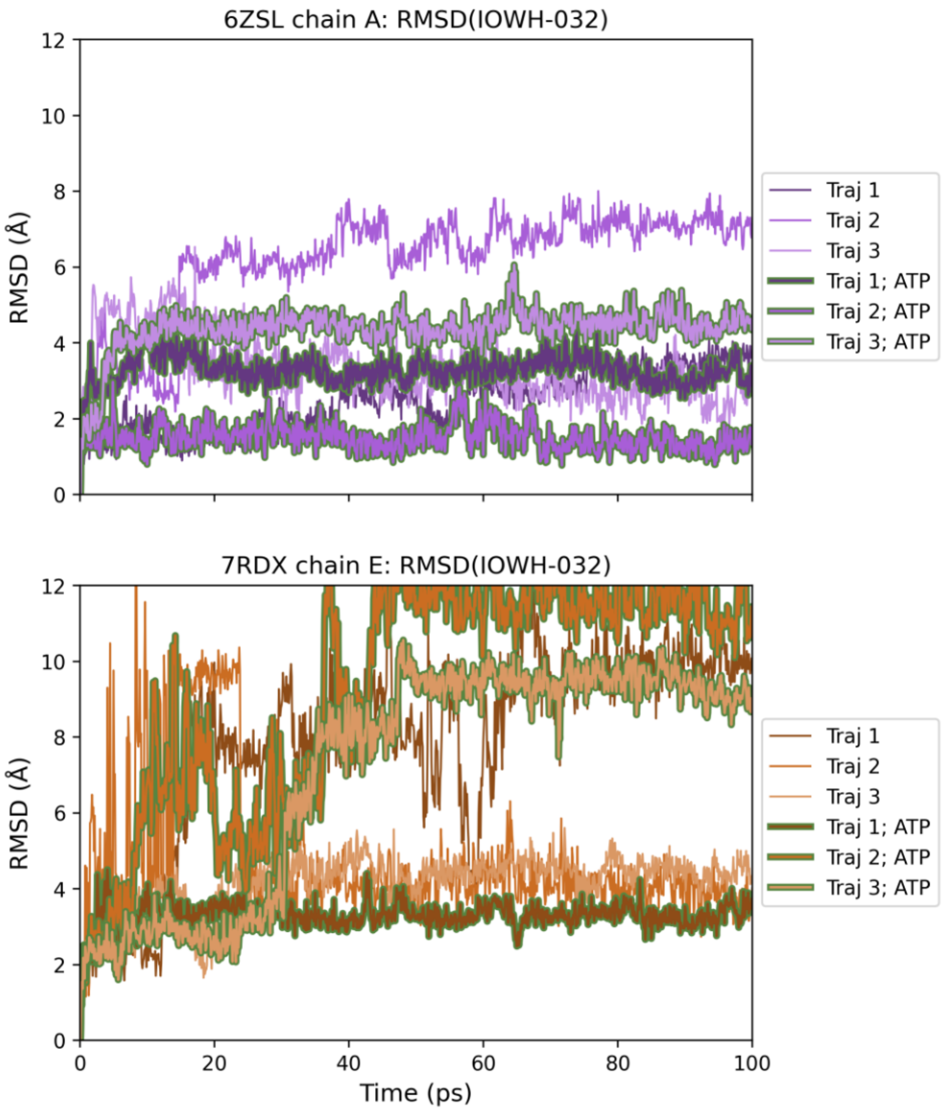

Figure S11. Heavy atom RMSD of IOWH-032 to the first frame after aligning each frame to the initial structure by using the protein structure heavy atoms as the reference. The 6ZSL chain A simulation is run with IOWH-032 bound in the RNA binding site and the 7RDX chain E simulation is run with IOWH-032 bound in the allosteric site. Trajectories with RMSD(IOWH-032) > 6 Å were discarded prior to the initial residue interaction analysis. Trajectory 1 of the 6ZSL chain A simulation, trajectory 1 and the first 30 ps of trajectory 2 of the 7RDX chain E simulations, and trajectories 2 and 3 of the 7RDX chain E with ATP bound simulations were discarded. The high RMSD observed for half of the allosterically bound trajectories suggest the instability of the compound at that site. In contrast, the lower RMSD observed for most of the RNA-site bound trajectories support binding of IOWH-032 to the RNA interaction site.

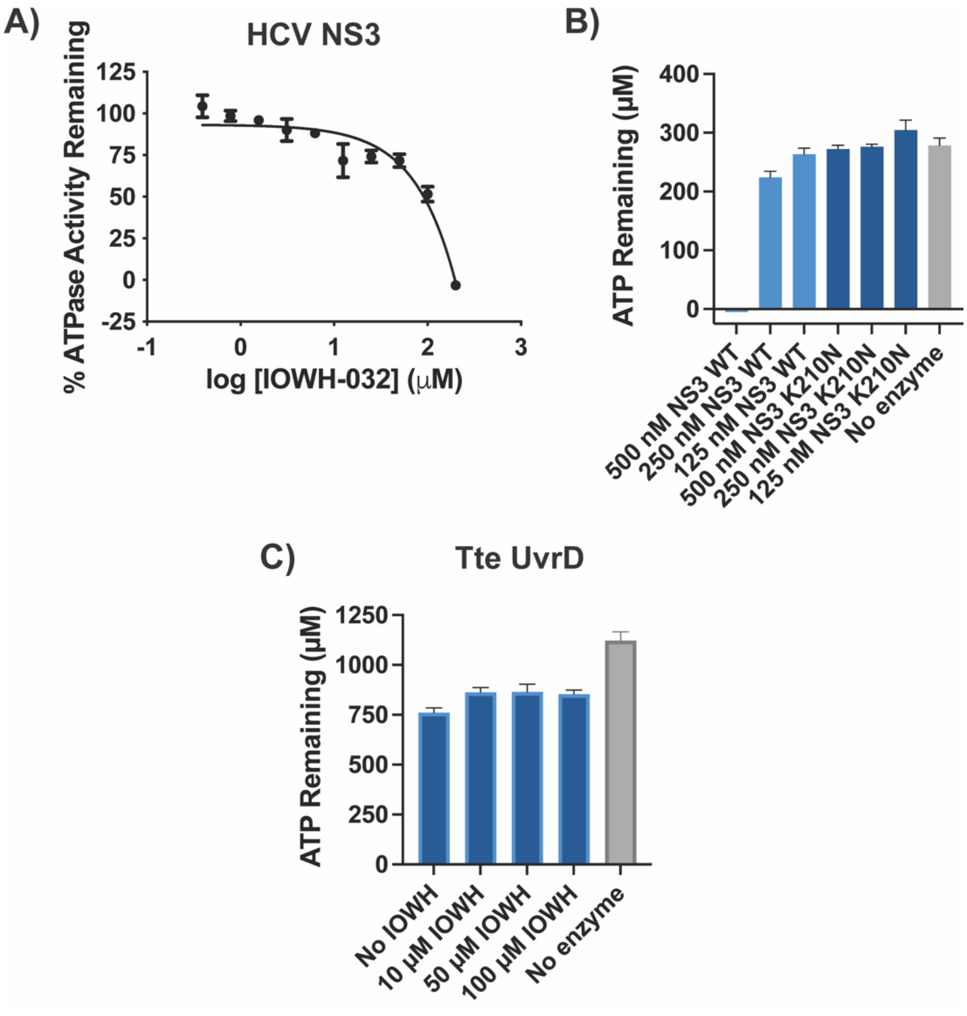

Figure S12. IOWH-032 does not inhibit other microbial helicases at micromolar concentrations. (A) Titration of 250 nM Hepatitis C Virus (HCV) RNA helicase NS3 with IOWH-032 and 250 μM ATP showed that ATPase activity decreases only when concentrations of IOWH-032 exceed 100 μM. NS3 (250 nM) was incubated with 250 μM ATP, and indicated concentrations of IOWH-032. (B) Analysis of indicated concentrations of WT NS3 versus a point mutant in a catalytic residue shows that ATP (250 μM) consumption relies on active NS3. (C) The consumption of ATP (1 mM) at 65 ºC by the bacterial DNA helicase *Thermoanaerobacter tengcongensis* (Tte) UvrD (3.02 nM) is not affected by increasing concentrations of IOWH-032. Error bars represent SD for n = 3.

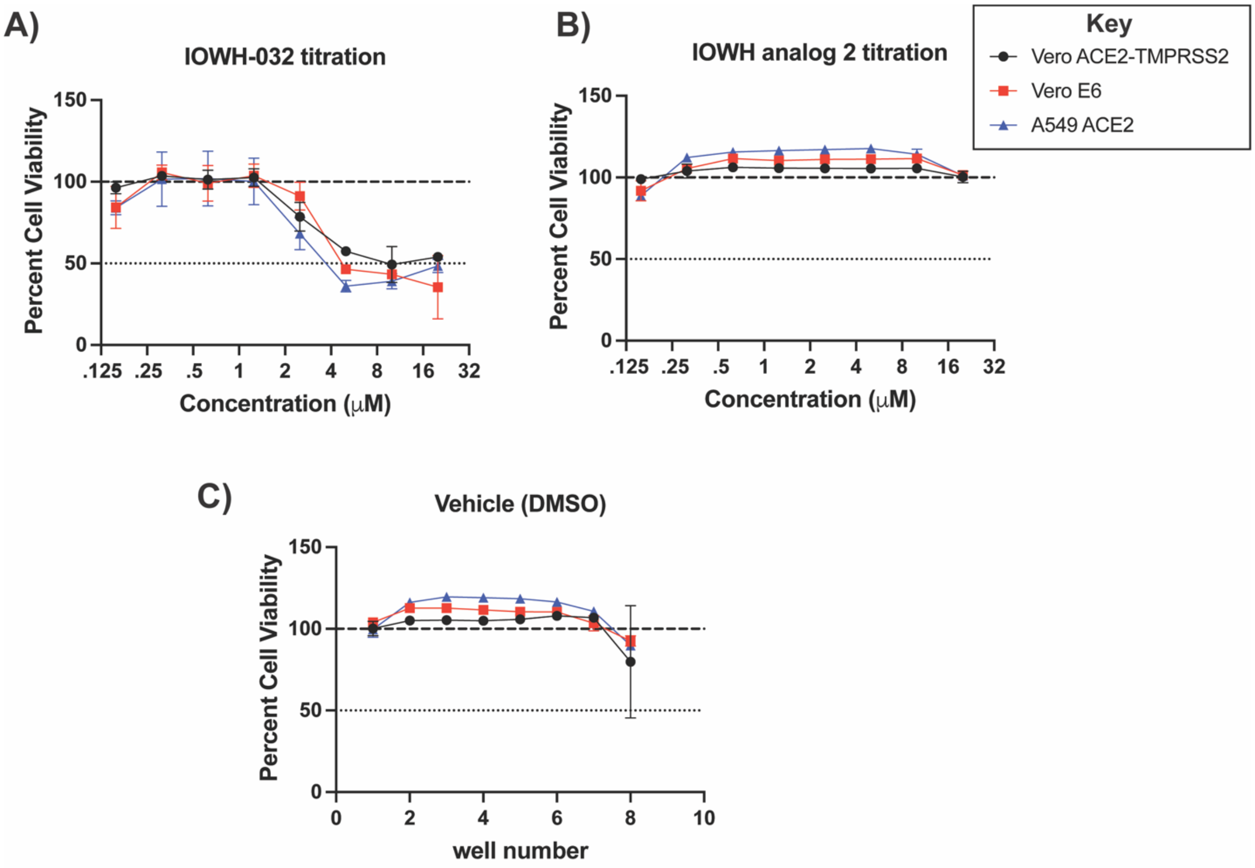

Figure S13. IOWH-032 is toxic against commonly used mammalian infection cell lines. (A-B) IOWH-032 and analog **2** were titrated into indicated cell lines in 0.1 % DMSO. (C) Constant concentration of vehicle (0.1% DMSO) was added to indicated cell lines across a multiwell plate row. Cells were incubated at 37 °C for t = 22 hr prior to measuring viability. Percent viability was calculated by comparison to cells that lacked any added inhibitor or vehicle. Vero cell lines are African Green Monkey kidney cells; the ACE2-TMPRSS2 subline overexpresses the human receptor and protease. A549 ACE2 cells are human lung adenocarcinoma cell lines that overexpress human ACE2. Error bars indicate SD for n = 3.

Supplemental Methods:

Production of plasmids by molecular cloning

All primers are listed in **Table S1**, plasmids are listed in **Table S2**, and strains are listed in **Table S3**. *E. coli* cells were grown in Luria-Bertani (LB) medium (Fisher) or Terrific Broth medium (IBI Scientific). Plasmids used in this work were constructed using overlap extension PCR[60] or obtained from AddGene. Site directed mutagenesis was performed using the Quickchange (Agilent) protocol with indicated primers and plasmids. Following mutagenesis, PCR products were DpnI (New England Biolabs) treated, purified using a PCR purification kit (Qiagen), and transformed into either Mach1 (Invitrogen) or DH5α (Invitrogen) competent cells. Following confirmation by DNA sequencing (Genewiz or Eton), plasmids were transformed into *E. coli* BL21 competent cells, unless otherwise noted (Novagen). For other overexpression cell lines used, see **Table S3**. COVID-SARS2 NSP13 was a gift from Opher Gileadi (Addgene plasmid # 159614 ; http://n2t.net/addgene:159614 ; RRID:Addgene_159614). pET His6 Sumo TEV LIC cloning vector (2S-T) was a gift from Scott Gradia (Addgene plasmid # 29711 ; http://n2t.net/addgene:29711 ; RRID:Addgene_29711). pET28a 6xHis PreScission SARS-CoV-2 nsp13 was a gift from Tarun Kapoor (Addgene plasmid # 159390 ; http://n2t.net/addgene:159390 ; RRID:Addgene_159390). pHYRS52 was a gift from Hideo Iwai (Addgene plasmid # 31122 ; http://n2t.net/addgene:31122 ; RRID:Addgene_31122)

Purification of pET28a 6xHis PreScission SARS-CoV-2 nsp13

Expression

The pET28a 6xHis PreScission SARS-CoV-2 nsp13 plasmid was received as a gift from the Kapoor Lab at The Rockefeller University. The expression of N-terminally His_6-tagged Nsp13 was conducted as previously described.[8] Briefly, the pET28a Nsp13 plasmid was transformed into *E. coli* Rosetta (DE3) cells (Novagen) and grown overnight at 37 °C on LB-agar plates containing kanamycin (25 µg/mL) and chloramphenicol (30 µg/mL). Isolated colonies were selected for small culture inoculation in 5 mL of LB media supplemented with kanamycin (25 µg/mL) and chloramphenicol (30 µg/mL) and grown with shaking overnight (37 °C, 200 RPM). These were then used to inoculate 500 mL of LB supplemented with kanamycin (25 µg/mL) and chloramphenicol (30 µg/mL) and grown to log-phase (OD_{600} = 0.6, optical density at 600 nm) with shaking (37 ºC, 200 RPM). Cultures were then induced with 0.2 mM isopropyl β-D-1-thiogalactopyranoside (IPTG) for 17 hours with shaking (16 ºC, 200 RPM). The cells were pelleted through centrifugation (3732 x g, 30 min) and stored at -80 ºC.

Purification

Cells from 500 mL cultures were resuspended with 15 mL of resuspension buffer (50 mM 2-(4-(2-hydroxyethyl)-1-piperazinyl)-ethanesulfonic acid (HEPES) pH 7.5, 500 mM NaCl, 4 mM $MgCl_2$, 5% (v/v) glycerol, 20 mM imidazole, 5 mM beta-mercaptoethanol (BME), 1 mM ATP, 1 mM phenylmethylsulfonyl fluoride (PMSF)) with protease inhibitors (SIGMAFAST Protease Inhibitor Cocktail Tablets, EDTA-Free). Additionally, lysozyme (0.1 mg/mL) and Dnase I (6.6 µg/mL) were added to the resuspended cells. The cells were rocked at 4 ºC for 1 hour and then lysed by sonication (Fisherbrand™ Model 120 Sonic Dismembrator) with an amplitude of 50% for 10 min (30 sec on and 30 sec off). Samples were then centrifuged (8,736 x g, 30 min, 4 ºC). The supernatant was incubated with 4 mL of washed Ni-NTA resin (Qiagen) on a gravity chromatography column for 30 min while rocking at 4 ºC. The protein was washed with 2 x 20 mL of wash buffer 1 (20 mM tris(hydroxymethyl)aminomethane-HCl (Tris-HCl), pH 8.0, 300 mM NaCl, 5% (v/v) glycerol, 20 mM imidazole, 1 mM BME, 1 mM ATP), and 2 x 20 mL of wash buffer 2 (20 mM Tris-HCl pH 8.0, 300 mM NaCl, 5% (v/v) glycerol, 30 mM imidazole, 1 mM BME, 1 mM ATP). Samples were eluted with 3 x 15 mL of elution buffer (20 mM Tris-HCl pH 8.0, 300 mM NaCl, 5% (v/v) glycerol, 250 mM imidazole, 1 mM BME, 1 mM ATP). Elution fractions were combined and dialyzed in 2L of dialysis buffer (50 mM HEPES-NaOH pH 7.5, 500 mM NaCl, 4 mM $MgCl_2$, 5%

(v/v) glycerol, 20 mM imidazole, 5 mM BME) overnight at 4 ºC using SnakeSkin Dialysis Tubing (Thermo Scientific, 10K MWCO).

Following dialysis, the sample was concentrated using an Amicon centrifugal unit (MilliporeSigma, 30K MWCO, 3732 x g RPM, 4 ºC). The protein was further purified via gel filtration on a HiLoad 16/600 Superdex 200 prep grade column (Cytiva) that was equilibrated with gel filtration buffer (25 mM HEPES-NaOH pH 7.5, 250 mM KCl, 1 mM $MgCl_2$, 1 mM TCEP) using an ÄKTA FPLC (GE HealthCare). 500 µL of protein was injected on the column and eluted over an isocratic flow of gel filtration buffer at 0.3 mL/min, over 1 column volume (CV). Purified Nsp13 was put into storage buffer (25 mM HEPES-NaOH pH 7.5, 250 mM KCl, 1 mM $MgCl_2$, 20% (v/v) glycerol, 1 mM tris(2-carboxyethyl)phosphine (TCEP)) and concentrated as described above. Samples were then aliquoted, flash frozen with liquid N_2, and stored at -80 ºC.

Purification of pNIC-ZB Covid-SARS2 Nsp13

Expression

The pNIC-ZB Covid-SARS2 Nsp13 plasmid was obtained from AddGene.[1] Expression and purification protocols followed previous literature.[9] In brief, the plasmid was transformed into *E. coli* BL21 (DE3) cells (Novagen) and grown overnight at 37 °C on LB-agar plates containing kanamycin (25 µg/mL). Isolated colonies were selected for small culture inoculation in 4 x 10 mL of LB supplemented with kanamycin (25 µg/mL) and grown with shaking overnight (37 °C, 200 RPM). These were then used to inoculate 4 x 1 L of Terrific Broth (IBI Scientific) supplemented with kanamycin (25 µg/mL) and grown to OD_{600} = 2-3 (optical density at 600 nm) with shaking (37 ºC, 200 RPM). Cultures were then induced with 0.3 mM isopropyl β-D-1-thiogalactopyranoside (IPTG) for 17 hours with shaking (18 ºC, 200 RPM). The cells were pelleted through centrifugation (3732 x g, 30 min) and stored at -80 ºC.

Purification

Cells from 4 L of culture were resuspended with 200 mL of resuspension buffer (50 mM HEPES-NaOH pH 7.5, 500 mM NaCl, 5% (v/v) glycerol, 10 mM imidazole, 0.5 mM TCEP) with protease inhibitors added (SIGMAFAST Protease Inhibitor Cocktail Tablets, EDTA-Free). Additionally, lysozyme (0.1 mg/mL) and Dnase I (6.6 µg/mL) were added to the resuspended cells. The cells were rocked at 4 ºC for 1 hour and then lysed using a homogenizer (Emulsiflex-C5, 10K-15K psi). Samples were then centrifuged (72,700 x g, 30 min, 4 ºC). The supernatant was split evenly into four gravity chromatography columns, each containing 8 mL of washed Ni-NTA resin (Qiagen), and incubated for 30 min while rocking at 4 ºC. Each column was washed with 40 mL of resuspension buffer, 25 mL of wash buffer (50 mM HEPES-NaOH pH 7.5, 500 mM NaCl, 5% (v/v) glycerol, 45 mM imidazole, 0.5 mM TCEP), 10 mL Hi-Salt buffer (50 mM HEPES-NaOH pH 7.5, 1 M NaCl, 5% (v/v) glycerol, 0.5 mM TCEP), and another 10 mL of wash buffer. The protein was then eluted off of each column using 10 mL of elution buffer B (50 mM HEPES-NaOH pH 7.5, 500 mM NaCl, 5% (v/v) glycerol, 300 mM imidazole, 0.5 mM TCEP).

The elution fractions were combined and immediately loaded onto 2 x 1 mL HiTrap SP HP columns (Cytiva) for cation exchange, using a 50 mL Super Loop (Cytiva), on the ÄKTA FPLC. Both were pre-equilibrated with elution buffer. After loading, the column was then washed with 2 CV of elution buffer B (1 mL/min) and proteins were eluted with 3 CV of Hi-Salt buffer (1 mL/min). The collected sample was incubated overnight while rocking at 4 ºC with TEV protease (1:40 mass ratio).

The sample was concentrated using an Amicon centrifugal unit (MilliporeSigma, 30K MWCO, 3732 x g, 4ºC). The protein was further purified via gel filtration on a HiLoad 16/600 Superdex 200 prep grade column (Cytiva) that was equilibrated with gel filtration buffer (50 mM HEPES-NaOH pH 7.5, 500 mM NaCl, 5 mM $MgCl_2$, 5% (v/v) glycerol, 0.5 mM TCEP) using the ÄKTA FPLC. 500 µL of protein was injected on the column and eluted over an isocratic flow of gel filtration buffer at 1 mL/min, over 1 CV. Purified Nsp13 was concentrated as described above. Samples were then aliquoted, flash frozen with liquid N_2, and stored at -80 ºC. Unless otherwise

noted, this construct was used for most experiments due to improved stability and yields relative to the initial Nsp13 construct (6xHis PreScission SARS-CoV-2 Nsp13), above.

For purification of Nsp13 mutants, due to the reduced stability of constructs, some steps were slightly changed. Namely, post-cation exchange chromatography, the protein was diluted with gel filtration buffer (50 mM HEPES-NaOH pH 7.5, 500 mM NaCl, 5 mM $MgCl_2$, 5% (v/v) glycerol, 0.5 mM TCEP) using a 1:5 dilution, and glycerol was added to increase the final glycerol percentage to 10% for the incubation with TEV Protease overnight.

Purification of pETHisSUMO HCV NS3 Helicase

Expression

A truncated form of the Hepatitis C NS3 helicase protein (GenBank No. AJ238799.1: 3918 to 5321) obtained from Twist Biosciences was cloned into a pETHis-SUMO expression vector.[2] Expression and purification protocols followed previous literature.[10] In brief, the plasmid was transformed into *E. coli* BL21 (DE3) cells (Novagen) and grown overnight at 37 °C on LB-agar plates containing carbenicillin (50 µg/mL). Isolated colonies were selected for small culture inoculation into 5 mL of LB media supplemented with carbenicillin (50 µg/mL) and grown with shaking overnight (37 °C, 200 RPM). These were then used to inoculate 500 mL of LB supplemented with carbenicillin (50 µg/mL) and grown to log-phase (OD_{600} = 0.6, optical density at 600 nm) with shaking (37 ºC, 200 RPM). Cultures were then induced with 0.5 mM IPTG for 17 hours with shaking (16 ºC, 200 RPM). The cells were pelleted through centrifugation (3732 x g, 30 min) and stored at -80 ºC.

Purification

Cells from a 500 mL culture were resuspended with 25 mL of resuspension buffer (25 mM HEPES-NaOH pH 7.4, 75 mM KCl, 10% (v/v) glycerol, 1 mM Dithiothreitol (DTT)) with protease inhibitors added (SIGMAFAST Protease Inhibitor Cocktail Tablets, EDTA-free). The cells were lysed using a homogenizer (Emulsiflex-C5, 10K-15K psi). Samples were then centrifuged (72,700 x g 30 min, 4 ºC). The supernatant was loaded onto a 5 mL HisTrap FF column (GE Healthcare), using a 50 mL Super Loop (Cytiva), on the ÄKTA FPLC. Both were pre-equilibrated with resuspension buffer. The method involved washing the column with the resuspension buffer (25 mM HEPES-NaOH pH 7.4, 75 mM KCl, 10% (v/v) glycerol, 1 mM DTT) and implementing an increasing gradient of elution buffer (25 mM HEPES-NaOH pH 7.4, 75 mM KCl, 10% (v/v) glycerol, 1 mM DTT, 300 mM imidazole) from 0-100% and monitoring elution absorbance at 280 nm. The collected NS3 fraction was incubated overnight at 4 ºC with Ulp1 protease (1:500 mass ratio)[11, 12], while dialyzing in 2 L of resuspension buffer in SnakeSkin Dialysis Tubing (Thermo Scientific, 10K MWKO). Following dialysis, the sample was loaded onto 2 x 1 mL HiTrap Q FF columns (GE Healthcare), again using the 50 mL superloop. Both were pre-equilibrated with low salt buffer (25 mM HEPES-NaOH pH 7.4, 10% (v/v) glycerol, 1 mM DTT) that lacked KCl. The method involved an increasing gradient of high salt buffer (25 mM HEPES-NaOH pH 7.4, 500 mM KCl, 10% (v/v) glycerol, 1 mM DTT) from 0-100% and monitoring elution absorbance at 280 nm. Fractions containing NS3 were dialyzed overnight in 2 L of resuspension buffer. The final dialyzed sample was concentrated as described above and loaded onto a HiLoad 16/600 Superdex 200 column that had been pre-equilibrated in resuspension buffer. 500 µL of protein was injected on the column and eluted over an isocratic flow of gel filtration buffer at 1 mL/min, over 1 CV. Final protein samples were collected, concentrated, aliquoted, flash frozen with liquid N_2, and stored at -80 ºC.

A point mutation of NS3 was expressed and purified in the same manner.

Nsp13 ATPase Inhibition Assays

For Nsp13 ATPase assays, reactions were prepared with indicated amounts of protein in Nsp13 ATPase Buffer (25 mM HEPES-NaOH pH 7.5, 50 mM NaCl, 5 mM $MgCl_2$, 1 mM DTT, 0.01% Tween-20, unless otherwise noted). Compound or DMSO vehicle was added, making the final DMSO concentration 10%. Reaction components were incubated at 25 ºC for 15-30 min and

then initiated with a final concentration of 250 μM ATP, unless otherwise noted, bringing the final reaction volume to 15 μL. Reactions were then quenched with 15 μL of 0.05% formic acid at the designated time point.

For Kinase Glo (Promega) analysis, quenched reactions (15 μL) were added to a 96-well half area opaque plate (Corning) with 15 μL of Kinase Glo reagent (Promega) and incubated for 45 min with shaking (25 ºC, 200 RPM). Plates were then analyzed by measuring luminescence on a plate reader (Molecular Devices, Spectra Max iD5, 1 s integration; or Molecular Devices, FlexStation 3) using SoftMax Pro 7.1 software. Concentrations of ATP remaining were calculated using an ATP standard curve in Microsoft Excel, and analyzed/graphed in GraphPad Prism 9 software. Normalized percent inhibitions were calculated based on reactions lacking compound with only vehicle (DMSO) added (0%), and reactions without Nsp13 or with a catalytically inactive mutant containing vehicle only (100%).

For ADP-Glo (Promega) analysis, quenched reactions (10 μL) were added to a 96-well half area opaque plate (Corning) with 10 μL of ADP-Glo reagent (Promega) and incubated for 45 min with shaking (25 ºC, 200 RPM). Kinase Detection Reagent (20 μL) (Promega) was then added to each well and incubated for an additional 45 min with shaking (25 ºC, 200 RPM). Plates were then analyzed by measuring luminescence on a plate reader (Molecular Devices, Spectra Max iD5, 1 s integration) using SoftMax Pro 7.1 software. Concentrations of ADP were calculated using an ATP-to-ADP standard curve in Microsoft Excel, and analyzed/graphed in GraphPad Prism 9 software. Normalized percent inhibitions were calculated based on reactions lacking compound with only vehicle (DMSO) added (0%), and reactions without Nsp13 containing vehicle only (100%).

For ATPase IC_{50} measurements, reactions were set-up as stated above but with varying concentrations of compound. Compound was serially diluted from the designated maximum concentration, using 2-fold dilutions, and added to designated reactions making the final DMSO concentration 10%. Reactions were initiated with the noted ATP concentrations. KinaseGlo reagents (Promega) were used as previously described to determine the ATP remaining for each reaction. Normalized percent inhibitions were calculated based on reactions with only vehicle (DMSO) added (0%), and reactions without Nsp13 containing vehicle (100%). The data was fit in Prism 9 using the following equation: $Y = 100/(1+10^{((LogIC_{50}-X)*HillSlope)})$. Hill slopes were allowed to vary to obtain the best fit and because Nsp13 is predicted to form a dimer, which suggests that compound:protein binding is likely not 1:1.

For ATPase kinetics assays, reactions were set-up as stated above but with varying [ATP]. ATP was serial diluted from 1 mM, using a 2-fold serial dilution. The reactions were then initiated using these varying concentrations of ATP. ADP-Glo reagents (Promega) were used as previously described to determine the [ADP] produced for each reaction. Michaelis-Menten parameters K_m and V_{max} were calculated by fitting initial rates (μM/sec) vs. [ATP] (μM) in Prism 9 with the following equation: $Y = V_{max}*X/(K_m+X)$. K_{cat} was calculated by dividing V_{max} by total [enzyme].

HCV NS3 ATPase Inhibition Assays

For NS3 ATPase assays, reactions were prepared with indicated amounts of protein in the ATPase Buffer B (25 mM 4-Morpholinepropanesulfonic acid (MOPS), pH 6.5, 1.25 mM $MgCl_2$, 5% DMSO, 50 μg/mL BSA, 0.01% Tween-20). Compound or DMSO was added, making the final DMSO concentration 5%. Reaction components were incubated at 23 ºC for 10 min and then reactions were initiated with 250 μM ATP, bringing the final reaction volume to 15 μL. Reactions were then quenched with 15 μL of 0.05% formic acid at the designated time point. Kinase Glo (Promega) was used as previously described to quantify ATP remaining. Plates were then analyzed by measuring luminescence on a plate reader (Molecular Devices, Spectra Max iD5, 1 s integration) using SoftMax Pro 7.1 software and analyzed/graphed in GraphPad Prism 9

software. Normalized percent inhibitions were calculated based on reactions with only vehicle (DMSO) added (0%), and reactions without Nsp13 containing vehicle only (100%).

For ATPase IC_{50} measurements, reactions were set-up as stated above but with varying concentration of compound. Compound was serially diluted from the designated maximum concentration, using 2-fold dilutions, and added to designated reactions making the final DMSO concentration 5%. Compounds were initiated with ATP. Kinase Glo reagents (Promega) were used as previously described to determine the ATP remaining for each reaction. Normalized percent inhibitions were calculated based on reactions with only vehicle (DMSO) added (0%), and reactions without NS3 containing vehicle only (100%). The data was fit in Prism 9 using the following equation: $Y = 100/(1+10^((LogIC_{50}-X)*HillSlope))$. Hill slopes were allowed to vary to obtain the best fit, as it was not known if the compound would bind with 1:1 stoichiometry.

Tte UvrD ATPase Inhibition Assays

Tte UvrD helicase protein was obtained from New England Biolabs (Cat. #M1202S) and the manufacturer's protocol was followed using their recommended assay conditions: 62.5 nM DNA (same DNA duplex as used in Nsp13 helicase inhibition assays), 1 mM ATP, 2 μL of Isothermal Amplification Buffer (NEB). Compound or DMSO was added, making the final DMSO concentration 5%. Reaction components were incubated at 65 ºC for 10 min and then initiated with 5 ng of Tte UvrD helicase, bringing the final reaction volume to 20 µL. Reactions were then quenched with 20 µL of 20 mM EDTA at the designated time point. Activity was quantified using the previously mentioned Kinase Glo reagents in a 384-well, flat bottom, opaque microplate (Grenier). Plates were then analyzed by measuring luminescence on a plate reader (Molecular Devices, Spectra Max iD5, 1 s integration) and analyzed/graphed in GraphPad Prism 9 software.

Nsp13 Helicase Inhibition Assays

FRET-based helicase assays were conducted with indicated amounts of protein in Nsp13 Helicase Buffer (20 mM HEPES-NaOH pH 7.5, 20 mM NaCl, 5 mM $MgCl_2$, 1 mM DTT, 0.01% Tween-20 unless otherwise noted) with 50 nM labeled DNA-duplex, unless otherwise noted. The labeled DNA duplex was obtained from Integrated DNA Technologies by pre-annealing Oligomer 1 (5'-/Cy3/CCTCGTAGGTACGCATGGATCCTCT-3') and Oligomer 2 (5'-GCTTGCATGCCTGCAGGTGCACTCTAGAGGATCCATGCGTACCTACGAGG/BHQ_2/-3'). These oligomers were annealed using a Bio-Rad Laboratories T100^{TM} Thermocycler (95 ºC for 5 minutes, -1ºC/cycle for 1 min/cycle (70 cycles), and then held at 4ºC). Compound or DMSO was added, making the final DMSO concentration 10%. Reaction components were mixed and incubated at 28 ºC for t = 15 min. Then 36 µL of reaction was added to a 96-well half area opaque plate (Corning) and the reaction was initiated with 40 mM ATP (4 µL), unless otherwise indicated, using auto-injectors in a plate reader (Molecular Devices, Spectra Max iD5), resulting in a final reaction volume of 40 µL and an initial ATP concentration of 4 mM, unless otherwise stated. Helicase unwinding was tracked at 28 ºC by measuring fluorescence (excitation: 515 nm and emission: 564 nm) every 0.5 s for 90 sec or over an indicated time course. The linear phase was analyzed and plotted using Prism 9 software.

For IC_{50} measurements, reactions were set-up as stated above but with varying concentration of compound. Compound was serially diluted from the designated maximum concentration, typically using 2-fold dilutions, and added to designated reactions lacking ATP making the final DMSO concentration 10%. Reactions were initiated with ATP using auto-injectors in the plate reader. Helicase unwinding was tracked as previously described. Normalized percent inhibition values were calculated using the RFU/sec of reactions with only vehicle (DMSO) added (0%), and the RFU/sec of reactions without Nsp13 containing vehicle only (100%). Each of the Nsp13 constructs were tested at concentrations that provides a measurable initial linear phase for helicase activity. The following concentrations were used: 25 nM: R21A, R21W, E142W; 12.5 nM: E341D, T380A, Y180A, E142A; 3.125 nM: P77L, R392C. The data was fit in Prism 9 using

the following equation: Y = 100/(1+10^((LogIC_{50}-X)*HillSlope)). Hill slopes were allowed to vary to obtain the best fit, as stated above.

For helicase kinetic analysis, the [labeled DNA-duplex] was varied as indicated. Michaelis-Menten parameters, K_M and V_{max}, were calculated by fitting initial rate (RFU/sec) vs. [DNA duplex] (nM) in Prism 9 with the following equation: Y = V_{max}*X/(K_m+X).

Fluorescence Polarization (FP) binding and displacement assays

FP assays were carried out by following published protocols.[13] The probe used for FP was a DNA duplex-FL similar to that used in the helicase assay, but lacking a quencher: Oligomer 1 (5'-/Fluorescein/CCTCGTAGGTACGCATGGATCCTCT-3') and Oligomer 2 (5'-GCTTGCATGCCTGCAGGTGCACTCTAGAGGATCCATGCGTACCTACGAGG-3'). These oligomers were annealed using a Bio-Rad Laboratories T100^{TM} Thermocycler (95 ºC for 5 minutes, -1ºC/cycle for 1 min/cycle (70 cycles), and then held at 4ºC). To quantify direct binding of probe to Nsp13, 20 or 100 nM of the duplex-FL was used with varying concentrations of Nsp13 construct. Both probe and protein were diluted in Helicase Buffer (20 mM HEPES-NaOH pH 7.5, 20 mM NaCl, 5 mM $MgCl_2$, 1 mM DTT, 0.01% Tween-20), along with 10% DMSO, in a 96-well half-area opaque plate (Corning). The plate was incubated with shaking (25 ºC, 200 RPM). The plates were read with a plate reader (Molecular Devices, Spectra Max iD5) equipped with excitation and emission filters at 485 nm and 535 nm, respectively, at t = 15 – 180 min. To determine the appropriate amount of Nsp13 to use in further competition experiments, the EC_{70} was calculated using the following equations: logEC_{70}=logEC_{70} - (1/HillSlope)*log(70/(100-70)) and Y=Bottom + (Top-Bottom)/(1+10^((LogEC_{70}-X)*HillSlope)).

For competition assays, the FP-duplex (20 or 100 nM) and Nsp13 construct (at the calculated EC_{70} value) were titrated with increasing concentrations of IOWH-032 or analog, as indicated in the figure caption. For **Figure 5C**, the following concentrations of protein were used for displacement experiments, which represent the 70% saturation values: WT (356 nM); Y180A (182 nM); R21A (124 nM); E142A (179 nM); T380A (227 nM).

The plates were rocked and measured using the same method and plate reader as described for the direct binding assays above. Data was analyzed in Prism with controls of duplex-FL alone and duplex-FL with saturating Nsp13 construct concentrations, and half-maximal effective concentration (EC_{50}) values were calculated for each with the equations: logEC_{50}=logEC_{50} - (1/HillSlope)*log(50/(100-50)) and Y=Bottom + (Top-Bottom)/(1+10^((LogEC_{50}-X)*HillSlope)). K_I values were then calculated from the EC_{50} and K_D values using previously described methods.[14]

Cytotoxicity assay against infection cell lines

Vero E6 ACE2-T2A-TMPRSS2 (BEI Resources, NR-54970), Vero E6 (ATCC #CRL-1586), and A549-ACE2 cells[15] (kind gift of Meike Dittmann) were cultured in 1x Dulbecco's modified Eagle's medium (Corning #10-013-CV) supplemented with 10% heat-inactivated FBS, 1x penicillin-streptomycin, and 2 mM L-glutamine and grown at 37 °C with 5% CO_2. Cells from each line were plated in tissue culture-treated black-walled and -bottomed 96 well plates with 1.5 x 10^4 cells in 100 µL of culture media per well. Plates were incubated overnight at 37 °C with 5% CO_2. The following day cells were washed with 1 x PBS (+Ca^{2+} +Mg^{2+}) and active compound, inactive compound, and DMSO vehicle control were added in triplicate, while remaining cells had no additives. All compounds were diluted in treatment media (1 x DMEM (Gibco #11-965-092) supplemented with 2% heat-inactivated FBS. Compounds were 2-fold serially diluted starting at 20 µM and ending at 156.25 nM (all in a final concentration of 0.1% DMSO when incubated with cells). Cells were incubated overnight (t = 22 hr) at 37 °C with 5% CO_2. The next day cells were fixed with 4% paraformaldehyde for 1 hour at room temperature then permeabilized with 3% bovine serum albumin in 1 x PBS with 0.1% Triton X-100 for 30 min at room temperature. Following permeabilization, cells were stained with DAPI at 1.25 µg/mL diluted in 3% BSA in 1 x

PBS for 1 hour at room temperature. Following staining, all plates were read on a CyTek CyTation 7 plate imager for cell counts. CC_{50} values were calculated in GraphPad Prism 9.5.1 using a nonlinear fit with variable slope.

Initial virtual screen

When the project began in 2020, the only structure available was a homolog to the SARS-CoV-2 Nsp13, the SARS-CoV-1 Nsp13 (PDB ID: 6JYT).[16] This structure shares a 99.8% sequence identity to the SARS-CoV-2 Nsp13, with a single point mutation difference at I150V at a site located away from the ATP and RNA binding sites.[17] Around 6,000 compounds from the Drug Repurposing Hub of the Broad Institute were first screened to the ATP binding site of this structure.[7]

RDKit version 2020.03.1[18] was used to read in the compounds in the SMILES format, add hydrogens, and generate an initial low energy 3D conformer for each compound using a distance geometry algorithm with MMFF94 force field.[19] OpenBabel 2.4.1 was then used to generate up to 10 conformers per compound using the genetic algorithm with the default options.[20] AlphaSpace was then used to perform fragment-centric topographical mapping of the SARS-CoV Nsp13 surface.[21] Two targetable binding pockets, the ATP and RNA binding sites, were identified as highly targetable binding sites. The OpenBabel-generated conformers were docked using flexible ligand docking with a fork of the Smina docking suite[22] that has Lin_F9 scoring function built-in[23] and retaining only the top 5 poses. The default docking parameters of Smina, such as exhaustiveness = 8, num_modes = 9, autobox_add = 4 were used for docking. The top 5 poses were retained, and 24 compounds were selected using thresholds of Lin_F9 score <= -8.5 kcal/mol, ligand efficiency <= -0.28, ligand coverage >= 0.85, ligand β-score <= -8.5 kcal/mol (**Figure S1**). Later, the Δ_{Lin_F9}XGB scoring function[24] was developed and used to rescore the top 5 docked poses. In the second screen, we replaced the Lin_F9 and ligand efficiency thresholds ΔLin_F9XGB score <= -9.0 kcal/mol and ligand efficiency <= -0.30. From this screen, 104 compounds were selected (**Figure S3**).

Blind ensemble docking

The hit compound IOWH-032 was blindly docked to the ensemble of SARS-CoV-2 receptor structures.

The ensemble of structures was parsed and analyzed using the beta version of the python package dist_analy (https://github.com/echen1214/dist_analy). The corresponding data and tutorials are available on (https://github.com/echen1214/nsp13_sbdd_md_data). The available 12 and 60 cyro-electron microscopy (cryo-EM) and X-ray crystallography structures, respectively, of SARS-CoV-2 Nsp13 as of 08/01/22 were retrieved from the Protein Data Bank using the pyPDB API [UniProt: P0DTD1].[25, 26] Further parsing and splitting of the structures resulted in 24 and 61 monomer chains from cryo-EM and X-ray crystallography, respectively. Cryo-EM structures of Nsp13 in the replication–transcription complex (RTC) is often observed with two protomers in complex with the RNA-dependent RNA-Polymerase (RdRp). We followed the nomenclature proposed by Hillen,[27] which defines $Nsp13_T$ (binding on the 'Thumb' side of the RdRp) and $Nsp13_F$ (binding on the 'Fingers' side of the RdRp). Next, a machine learning protocol using Scikit-learn was applied to analyze the conformational ensemble of the structure.[28] A pairwise residue–residue shortest distance matrix was calculated for each monomer chain to represent the protein structure. Then, two unsupervised machine learning algorithms were used to analyze the structural ensemble. Principal Component Analysis (PCA) was used to visualize the variance of the cryo-EM structural features set along the principal components.[29] The data points were projected on the first two principal components which comprise 92.6% of the explained variance ratio. Hierarchical Density-Based Spatial Clustering of Applications with Noise (HDBSCAN) was used to cluster the projected data points into three clusters.[30] The X-ray crystallography structures are then projected onto the PCA plot (**Figure S8, Table S6**). The resulting plot and clustering

underscore the conformational diversity of the cryo-EM structures in contrast to the conformational homogeneity of the resolved X-ray crystal structures. Cluster 1 (green) was characterized by $Nsp13_T$ monomer in the open state, whereby it is proposed the RNA was not engaged with the helicase and allowing the RdRp to perform its translocation and RNA synthesis activity. Cluster 2 (red) was characterized by $Nsp13_T$ monomer in the engaged and swivel conformations, whereby it was proposed RdRp was in the backtracking complex. Cluster 2 (cyan) was represented by the $Nsp13_F$ or *apo* $Nsp13_T$ conformations. When projected on the PCA plot, all the X-ray crystal structures were located near cluster 2. Each cryo-EM and X-ray crystal receptor conformation is then prepared by using the pdb2pqr30 v3.5.2 to add hydrogens and rebuild any missing side chains, and MGLTools 1.5.6 `prepare_receptor4.py` to add Gasteiger charges and AutoDock Vina types and to remove the non-polar hydrogens to the receptor.[31, 32]

Starting from the SMILES string of IOWH-032, Open Babel 3.1.0 was used to add hydrogens and generate 20 conformers using the using the genetic algorithm with the default options.[20] Each ligand conformer was prepared by using Meeko 0.3.3 to add Gasteiger charges and AutoDock Vina types, and remove the non-polar hydrogens. Each conformer was blindly docked to the ensemble of receptors with a fork of the Smina docking suite[8] that built in the Lin_F9 scoring function.[23]

Each docking score was re-scored using Δ_{Lin_F9}XGB to provide more accurate binding affinity predictions.[24] Δ_{Lin_F9}XGB used an ensemble of eXtreme Gradient Boosting (XGBoost) trees to predict a correction to the initial Lin_F9 score. Δ_{Lin_F9}XGB required a multitude of features to input into XGBoost version 1.2.0. The 48 Vina features were generated using AutoDock Vina 1.1.2 The SASA features were calculated with MSMS 2.6.1. The β-cluster features were calculated using AlphaSpace 2.0 and the Vina scoring function. The ligand features were generated with RDKit 2020.09.3. The sum of the Lin_F9 score and the XGBoost corrections results in the final binding affinity prediction (pK_d). The top 5 scoring poses for each receptor were used for further analysis.

To analyze the binding location of IOWH-032 following ensemble docking, the shortest ligand–receptor distance vector for each pose was calculated. This vector characterized the location of the docking pose relative to the receptor. These vectors were then analyzed with PCA and HDBSCAN. Here the docking poses and corresponding binding scores were clustered into three distinct binding sites, the ATP, RNA and one allosteric binding site (**Figure S9A, Table S7).** The RNA binding pose could be distinguished into two sub-clusters RNA #1 and #2. The RNA #1 pose bound in the center of the RNA binding pocket at the core of the protein. The RNA #2 pose bound at the proximal region of the RNA binding site (with respect to the view seen in **Figure 1B**). IOWH-032 was only found docked to the allosteric binding site when docked to 7RDX chain E from cluster 1. The cluster 1 structures reflected the 1B-domain swiveled to be near the Zinc-finger domain and created the allosteric binding pocket that was unique to this cluster of structures.

Computational Methods

Molecular dynamics (MD) simulations were performed for IOWH-032 bound to Nsp13 helicase with and without ATP bound. The corresponding parameter files are available on (https://github.com/echen1214/nsp13_sbdd_md_data). The starting poses of the IOWH-032 and Nsp13 complex were determined following the blind docking of IOWH-032 to the structural ensemble of Nsp13 (**Figure S8**). The ensemble docking results suggested three sites that IOWH-032 could bind: ATP, RNA, and allosteric sites. We did not run MD simulations of the ATP site because initial experiments indicated that IOWH-032 did not bind at that site (**Figure 4A**). The allosteric site-bound state was represented by the top 1 ranking Δ_{Lin_F9}XGB pose docked to the $Nsp13_T$ protomer taken from the class IV (1B-open) cryo-EM structure of the replication-transcription complex [PDB ID: 7RDX; chain E].[33] This pose was found to be clustered to the allosteric cluster. The resolved ADP molecule was modified to ATP by providing the ATP

parameter file and using *tleap* to rebuild the missing phosphate.[34, 35] The RNA site-bound state was represented by the top 1 ranking Δ_{Lin_F9}XGB score pose docked to the Nsp13 monomer taken from chain A of the *apo* Nsp13 structure [PDB ID: 6ZSL; chain A].[9] This pose was found to be clustered to the RNA #1 cluster. The ATP and Mg^{2+} was modelled into this structure by aligning the AMP-PNP- and Mg^{2+}- bound Nsp13 (PDB ID: 7NN0)[9] and modifying the amide between the *β*- and *γ*-phosphates in AMP-PNP to oxygen. The missing loop regions were remodeled using Modeller.[36]

The system preparation, minimization and equilibration followed the methods reported by Weber and McCullagh[37] and are summarized below. The protein was modelled using ff14SB parameters[38], and ATP[34] and Mg^{2+} [39] were modelled using parameters obtained from the AMBER parameter database. Each individual IOWH-032 molecule pose was parameterized. First, Open Babel 3.1.0 was used to protonate the ligand molecule.[20] Then, the restrained electrostatic potential (RESP) charges of the pose was determined in three steps using Gaussian 16, Revision A.03 using B3LYP/6-31G* functional/basis set and antechamber.[40, 41] The pose was first geometry optimized and then the electronic potential was calculated with Merz-Singh-Kollman scheme.[42, 43] Finally, antechamber was used to calculate the RESP charges and any missing bonded parameters were parameterized with GAFF2. The protein structures were protonated using the H++ 4.0 webserver at pH 7.[44, 45] All crystallographic waters and zinc ions were retained. The Nsp13 zinc binding domain contains three zinc binding pockets. The three zincs and neighboring residues within 2.8 Å of the zinc were parameterized in CYM–CYM–CYM–CYM, CYM-CYM-CYM-HID, and CYM-CYM-HID-HIE environments using MCPB.py version 7.0 tool.[46] TIP3P water was added to each system with at least a 12 Å buffer. Na^+ and Cl^- ions were added to neutralize the charge and provide a 0.1 M salt concentration using the SLTCAP webserver.[47]

All-atom, explicit solvent MD simulations for the two initial starting poses of the IOWH-032, ATP, Nsp13 complex were performed using the GPU-enabled AMBER22.03 software.[35] Hydrogen atoms were constrained using the SHAKE algorithm.[48] Direct nonbonding interactions were cut off at 12, and long-range electrostatic interactions were modeled using the particle-mesh Ewald treatment.[49] Each simulation in the NPT ensemble was run with the Monte Carlo barostat set to 1 atm, Langevin thermostat set to 300 K and integration time step of 2 fs.

Each system followed the same minimization, equilibration, and production protocols. The system was minimized in 10 stages of 2000 steps of steepest descent minimization with varying harmonic restraints. The first stage used 500 kcal mol^{-1} $Å^{-2}$ restraints on all protein, ATP, and ligand atoms. In the next 4 stages, the restraint on the protein side chains was progressively lowered to 10.0, 1.0, 0.1, and 0.0 kcal mol^{-1} $Å^{-2}$. In the final 5 stages, the restraint on the protein backbone, ATP, and IOWH-032 ligand was progressively lowered to 50.0, 5.0, 1.0, 0.1, and 0.0 kcal mol^{-1} $Å^{-2}$. This minimized system spawned a triplicate of simulation that were equilibrated and then run separately. The system was heated from 0 to 300 K with a harmonic restraint of 40 kcal mol^{-1} $Å^{-2}$ on all protein, ATP, and ligand atoms. The pressure was then equilibrated in 7 stages. First, a 1 ns of NVT was run with the same harmonic restraints. In the following 6 pressure equilibration stages of 200 ps each, the restraint was reduced to 20.0, 10.0, 5.0, 1.0, 0.1, and 0 kcal mol^{-1} $Å^{-2}$. Finally, a conventional simulation was run for 100 ns with a snapshot saved at every 0.1 ns. This output was then used for analysis of the potential contacts between the IOWH-032 and Nsp13 at each site.

Analysis of MD simulations to predict residues that interact with IOWH-032

Analyses of the conventional MD simulations to select the residues for alanine/tryptophan mutations were performed using the CPPTRAJ V6.18.1 tool and the MMPBSA.py version 14.0 tools.[46, 50] Trajectories and frames that did not retain the IOWH-032 molecule in the binding pocket were discarded from further analysis. This was determined by aligning each frame to the starting frame and calculating the ligand heavy atom RMSD with the \`rmsd\` command. The trajectories with many frames with RMSD > 6 Å were discarded (**Figure S11**). Removing these trajectories

prioritized the determination of the residues that would be interacting with IOWH-032 during the analysis. This analysis assumes that removal of the side chain does not affect the structure.

To select residues for mutation, the side chain contact frequency (< 4.5 Å) with the ligand and the side chain energetic contributions to binding were measured per residue. The side chain contact frequency was measured using the `nativecontacts` command. The side chain energetic contributions to binding were calculated by performing binding free energy calculations using the MMPBSA.py tool.[51] This tool performed Molecular Mechanics-Poisson–Boltzmann (or Generalized Born) Surface Area (MM-PB/GBSA) calculations and relies on the end-points of the thermodynamic cycle to calculate binding free energies.[52-54] The calculations were carried out using "single-trajectory" protocol on all frames.[51] This protocol stripped the trajectory to create bound/unbound and solvated/un-solvated ensembles. The polar solvation free energy contribution was determined by solving the PB equation using the classical nonpolar solvent model (*inp* = 1) and atomic radii according to the parameter file[54] or by applying the "OBC" GB method using mbondi2 radii (*igb* = 5).[55] Both were computed at 100 mM ionic strength and with dielectric constant of 1 and 80 for the interior and exterior of the solute, respectively. The nonpolar solvation free energy contribution was calculated with the solvent accessible surface area (SASA) method.[56] The change in conformational entropy was neglected. The per residue contribution of the side chain to the free energy was calculated for both the GB and PB models.[57] The scheme that added the 1–4 terms to the internal potential terms (*idecomp* = 1) was used. This analysis is referenced in the main text as "per-residue side chain binding energy decomposition".

The residues that retained side chain contacts frequency > 0.9 and had an absolute side chain energetic contribution in at least one trajectory > 0.75 kcal/mol were selected. Of this subset of residues, five residues from each binding site that interacted with the ligand across the binding interface and range in polarity were selected for experimental mutagenesis.

Mutated Nsp13 simulation experiments

The mutations Y180A, E142A and E142W were introduced in the simulations to confirm the observed effect of mutation on binding. The ChimeraX `swapaa` command was used to mutate these residues to the desired side chain through sampling conformations from the Dunbrack rotamer library.[58, 59] To determine the initial pose of E142W–IOWH-032 complex, IOWH-032 was re-docked to the E142W structure and the top $\Delta_{\text{Lin_F9}}$XGB pose with IOWH-032 in the RNA site was selected. Otherwise, the force field parameters, system setup, minimization, equilibration, and production used for the wild-type complex were used in these simulations. No trajectories were removed during this analysis and ATP-*apo* state simulations were not run.

The binding free energies of IOWH-032 to the Nsp13–ATP complex (ΔG^{WT}) were determined with MM-PBSA and MM-GBSA calculations. These calculations were performed on each replicate using the "single-trajectory" protocol and following the same parameters as described above in the "Analysis of MD simulations to predict residues that interact with IOWH-032" section. The difference in binding energies between wildtype and mutants are calculated by

$$\Delta\Delta G = \Delta G^{MUT} - \Delta G^{WT}$$

References:

(1) COVID-SARS2 NSP13 was a gift from Opher Gileadi (Addgene plasmid # 159614 ; http://n2t.net/addgene:159614 ; RRID:Addgene_159614).
(2) pET His6 Sumo TEV LIC cloning vector (2S-T) was a gift from Scott Gradia (Addgene plasmid # 29711 ; http://n2t.net/addgene:29711 ; RRID:Addgene_29711).
(3) pET28a 6xHis PreScission SARS-CoV-2 nsp13 was a gift from Tarun Kapoor (Addgene plasmid # 159390 ; http://n2t.net/addgene:159390 ; RRID:Addgene_159390).
(4) pHYRS52 was a gift from Hideo Iwai (Addgene plasmid # 31122 ; http://n2t.net/addgene:31122 ; RRID:Addgene_31122)
(5) Bloom JD, Neher RA. Fitness effects of mutations to SARS-CoV-2 proteins. *Virus Evol.* **2023**, *9* (2), vead055.
(6) Hadfield, J.; Megill, C.; Bell, S. M.; Huddleston, J.; Potter, B.; Callender, C.; Sagulenko, P.; Bedford, T.; Neher, R. A. Nextstrain: real-time tracking of pathogen evolution. *Bioinformatics* **2018**, *34* (23), 4121-4123.
(7) Corsello, S. M.; Bittker, J. A.; Liu, Z.; Gould, J.; McCarren, P.; Hirschman, J. E.; Johnston, S. E.; Vrcic, A.; Wong, B.; Khan, M.; et al. The Drug Repurposing Hub: a next-generation drug library and information resource. *Nat. Med.* **2017**, *23* (4), 405-408.
(8) Chen, J.; Malone, B.; Llewellyn, E.; Grasso, M.; Shelton, P. M. M.; Olinares, P. D. B.; Maruthi, K.; Eng, E. T.; Vatandaslar, H.; Chait, B. T.; et al. Structural Basis for Helicase-Polymerase Coupling in the SARS-CoV-2 Replication-Transcription Complex. *Cell* **2020**, *182* (6), 1560-1573.e1513.
(9) Newman, J. A.; Douangamath, A.; Yadzani, S.; Yosaatmadja, Y.; Aimon, A.; Brandão-Neto, J.; Dunnett, L.; Gorrie-Stone, T.; Skyner, R.; Fearon, D.; et al. Structure, mechanism and crystallographic fragment screening of the SARS-CoV-2 NSP13 helicase. *Nature Commun.* **2021**, *12* (1).
(10) Yang, N.; Sun, C.; Zhang, L.; Liu, J.; Song, F. Identification and Analysis of Novel Inhibitors against NS3 Helicase and NS5B RNA-Dependent RNA Polymerase from Hepatitis C Virus 1b (Con1). *Front. Microbiol.* **2017**, *8*.
(11) Uehara, T.; Parzych, K. R.; Dinh, T.; Bernhardt, T. G. Daughter cell separation is controlled by cytokinetic ring-activated cell wall hydrolysis. *EMBO Rep.* **2010**, *29* (8), 1412-1422.
(12) Muona, M.; Aranko, A. S.; Iwai, H. Segmental isotopic labelling of a multidomain protein by protein ligation by protein trans-splicing. *Chembiochem* **2008**, *9* (18), 2958-2961.
(13) Hosfelt, J.; Richards, A.; Zheng, M.; Adura, C.; Nelson, B.; Yang, A.; Fay, A.; Resager, W.; Ueberheide, B.; Glickman, J. F.; et al. An allosteric inhibitor of bacterial Hsp70 chaperone potentiates antibiotics and mitigates resistance. *Cell Chem. Biol.* **2022**, *29* (5), 854-869 e859.
(14) Rossi, A. M.; Taylor, C. W. Analysis of protein-ligand interactions by fluorescence polarization. *Nat. Protoc.* **2011**, *6* (3), 365-387.
(15) de Vries, M.; Mohamed, A. S.; Prescott, R. A.; Valero-Jimenez, A. M.; Desvignes, L.; O'Connor, R.; Steppan, C.; Devlin, J. C.; Ivanova, E.; Herrera, A.; et al. A comparative analysis of SARS-CoV-2 antivirals characterizes 3CL(pro) inhibitor PF-00835231 as a potential new treatment for COVID-19. *J. Virol.* **2021**, *95* (7).
(16) Jia, Z.; Yan, L.; Ren, Z.; Wu, L.; Wang, J.; Guo, J.; Zheng, L.; Ming, Z.; Zhang, L.; Lou, Z.; et al. Delicate structural coordination of the Severe Acute Respiratory Syndrome coronavirus Nsp13 upon ATP hydrolysis. *Nucleic Acids Res.* **2019**, *47* (12), 6538-6550.
(17) Mirza, M. U.; Froeyen, M. Structural elucidation of SARS-CoV-2 vital proteins: Computational methods reveal potential drug candidates against main protease, Nsp12 polymerase and Nsp13 helicase. *J. Pharm. Anal.* **2020**, *10* (4), 320-328.
(18) RDKit: Open-source cheminformatics. https://www.rdkit.org. **2020**.
(19) Tosco, P.; Stiefl, N.; Landrum, G. Bringing the MMFF force field to the RDKit: implementation and validation. *J. Cheminf.* **2014**, *6* (1), 37.

(20) O'Boyle, N. M.; Banck, M.; James, C. A.; Morley, C.; Vandermeersch, T.; Hutchison, G. R. Open Babel: An open chemical toolbox. *J. Cheminf.* **2011**, *3* (1), 33.
(21) Rooklin, D.; Wang, C.; Katigbak, J.; Arora, P. S.; Zhang, Y. AlphaSpace: Fragment-Centric Topographical Mapping To Target Protein–Protein Interaction Interfaces. *J. Chem. Inf. Model.* **2015**, *55* (8), 1585-1599.
(22) Koes, D. R.; Baumgartner, M. P.; Camacho, C. J. Lessons Learned in Empirical Scoring with smina from the CSAR 2011 Benchmarking Exercise. *J. Chem. Inf. Model.* **2013**, *53* (8), 1893-1904.
(23) Yang, C.; Zhang, Y. Lin_F9: A Linear Empirical Scoring Function for Protein–Ligand Docking. *J. Chem. Inf. Model.* **2021**, *61* (9), 4630-4644.
(24) Yang, C.; Zhang, Y. Delta Machine Learning to Improve Scoring-Ranking-Screening Performances of Protein–Ligand Scoring Functions. *J. Chem. Inf. Model.* **2022**, *62* (11), 2696-2712.
(25) Gilpin, W. PyPDB: a Python API for the Protein Data Bank. *Bioinformatics* **2015**, *32* (1), 159-160.
(26) Berman, H. M.; Westbrook, J.; Feng, Z.; Gilliland, G.; Bhat, T. N.; Weissig, H.; Shindyalov, I. N.; Bourne, P. E. The Protein Data Bank. *Nucleic Acids Res.* **2000**, *28* (1), 235-242.
(27) Hillen, H. S. Structure and function of SARS-CoV-2 polymerase. *Curr. Opin. Virol.* **2021**, *48*, 82-90.
(28) Pedregosa, F.; Varoquaux, G.; Gramfort, A.; Michel, V.; Thirion, B.; Grisel, O.; Blondel, M.; Prettenhofer, P.; Weiss, R.; Dubourg, V.; et al. Scikit-learn: Machine Learning in Python. *J. Mach. Learn. Res.* **2011**, *12*, 2825–2830.
(29) Jolliffe, I. T.; Cadima, J. Principal component analysis: a review and recent developments. *Philos. Trans. R. Soc. A* **2016**, *374* (2065), 20150202.
(30) Campello, R. J. G. B.; Moulavi, D.; Sander, J.. Density-based Clustering Based on Hierarchical Density Estimates. In *LNCS*; Lecture Notes in Computer Science, 2013; pp 160–172.
(31) Jurrus, E.; Engel, D.; Star, K.; Monson, K.; Brandi, J.; Felberg, L. E.; Brookes, D. H.; Wilson, L.; Chen, J.; Liles, K.; et al. Improvements to the APBS biomolecular solvation software suite. *Protein Sci.* **2018**, *27* (1), 112-128.
(32) Morris, G. M.; Huey, R.; Lindstrom, W.; Sanner, M. F.; Belew, R. K.; Goodsell, D. S.; Olson, A. J. AutoDock4 and AutoDockTools4: Automated docking with selective receptor flexibility. *J. Comput. Chem.* **2009**, *30* (16), 2785-2791.
(33) Chen, J.; Wang, Q.; Malone, B.; Llewellyn, E.; Pechersky, Y.; Maruthi, K.; Eng, E. T.; Perry, J. K.; Campbell, E. A.; Shaw, D. E.; et al. Ensemble cryo-EM reveals conformational states of the nsp13 helicase in the SARS-CoV-2 helicase replication–transcription complex. *Nat. Struct. Mol. Biol.* **2022**, *29* (3), 250-260.
(34) Meagher, K. L.; Redman, L. T.; Carlson, H. A. Development of polyphosphate parameters for use with the AMBER force field. *J. Comput. Chem.* **2003**, *24* (9), 1016-1025.
(35) Case, D. A.; Aktulga, H. M.; Belfon, K.; Ben-Shalom, I. Y.; Berryman, J. T.; Brozell, S. R.; Cerutti, D. S.; T.E. Cheatham, I.; Cisneros, G. A.; Cruzeiro, V. W. D.; et al. Amber 2023. University of California, San Francisco, 2023.
(36) Šali, A.; Blundell, T. L. Comparative Protein Modelling by Satisfaction of Spatial Restraints. *J. Mol. Biol.* **1993**, *234* (3), 779-815.
(37) Weber, R.; McCullagh, M. Role of ATP in the RNA Translocation Mechanism of SARS-CoV-2 NSP13 Helicase. *J. Phys. Chem. B.* **2021**, *125* (31), 8787-8796.
(38) Maier, J. A.; Martinez, C.; Kasavajhala, K.; Wickstrom, L.; Hauser, K. E.; Simmerling, C. ff14SB: Improving the Accuracy of Protein Side Chain and Backbone Parameters from ff99SB. *J. Chem. Theory Comput.* **2015**, *11* (8), 3696-3713.
(39) Allnér, O.; Nilsson, L.; Villa, A. Magnesium Ion–Water Coordination and Exchange in Biomolecular Simulations. *J. Chem. Theory Comput.* **2012**, *8* (4), 1493-1502.

(40) Wang, J.; Wang, W.; Kollman, P. A.; Case, D. A. Automatic atom type and bond type perception in molecular mechanical calculations. *J. Mol. Graph.* **2006**, *25* (2), 247-260.
(41) *Gaussian 16 Rev. C.01*; Wallingford, CT, 2016.
(42) Singh, U. C.; Kollman, P. A. An approach to computing electrostatic charges for molecules. *J. Comput. Chem.* **1984**, *5* (2), 129-145.
(43) Besler, B. H.; Merz Jr., K. M.; Kollman, P. A. Atomic charges derived from semiempirical methods. *J. Comput. Chem.* **1990**, *11* (4), 431-439.
(44) Anandakrishnan, R.; Aguilar, B.; Onufriev, A. V. H++ 3.0: automating pK prediction and the preparation of biomolecular structures for atomistic molecular modeling and simulations. *Nucleic Acids Res.* **2012**, *40* (W1), W537-W541.
(45) Myers, J.; Grothaus, G.; Narayanan, S.; Onufriev, A. A simple clustering algorithm can be accurate enough for use in calculations of pKs in macromolecules. *Proteins* **2006**, *63* (4), 928-938.
(46) Li, P.; Merz, K. M., Jr. MCPB.py: A Python Based Metal Center Parameter Builder. *J. Chem. Inf. Model.* **2016**, *56* (4), 599-604.
(47) Schmit, J. D.; Kariyawasam, N. L.; Needham, V.; Smith, P. E. SLTCAP: A Simple Method for Calculating the Number of Ions Needed for MD Simulation. *J. Chem. Theory Comput.* **2018**, *14* (4), 1823-1827.
(48) Ryckaert, J.-P.; Ciccotti, G.; Berendsen, H. J. C. Numerical integration of the cartesian equations of motion of a system with constraints: molecular dynamics of n-alkanes. *J. Comput. Phys.* **1977**, *23* (3), 327-341.
(49) Darden, T.; York, D.; Pedersen, L. Particle mesh Ewald: An N·log(N) method for Ewald sums in large systems. *J. Chem. Phys.* **1993**, *98* (12), 10089-10092.
(50) Roe, D. R.; Cheatham, T. E., III. PTRAJ and CPPTRAJ: Software for Processing and Analysis of Molecular Dynamics Trajectory Data. *J. Chem. Theory. Comput.* **2013**, *9* (7), 3084-3095.
(51) Miller, B. R., III; McGee, T. D., Jr.; Swails, J. M.; Homeyer, N.; Gohlke, H.; Roitberg, A. E. MMPBSA.py: An Efficient Program for End-State Free Energy Calculations. *J. Chem. Theory. Comput.* **2012**, *8* (9), 3314-3321.
(52) Wang, E.; Sun, H.; Wang, J.; Wang, Z.; Liu, H.; Zhang, J. Z. H.; Hou, T. End-Point Binding Free Energy Calculation with MM/PBSA and MM/GBSA: Strategies and Applications in Drug Design. *Chem. Rev.* **2019**, *119* (16), 9478-9508.
(53) Kollman, P. A.; Massova, I.; Reyes, C.; Kuhn, B.; Huo, S.; Chong, L.; Lee, M.; Lee, T.; Duan, Y.; Wang, W.; et al. Calculating Structures and Free Energies of Complex Molecules: Combining Molecular Mechanics and Continuum Models. *Acc. Chem. Res.* **2000**, *33* (12), 889-897.
(54) Srinivasan, J.; Cheatham, T. E.; Cieplak, P.; Kollman, P. A.; Case, D. A. Continuum Solvent Studies of the Stability of DNA, RNA, and Phosphoramidate−DNA Helices. *J. Am. Chem. Soc.* **1998**, *120* (37), 9401-9409.
(55) Onufriev, A.; Bashford, D.; Case, D. A. Exploring protein native states and large-scale conformational changes with a modified generalized born model. *Proteins.* **2004**, *55* (2), 383-394.
(56) Gilson, M. K.; Honig, B. Calculation of the total electrostatic energy of a macromolecular system: Solvation energies, binding energies, and conformational analysis. *Proteins.***1988**, *4* (1), 7-18.
(57) Gohlke, H.; Kiel, C.; Case, D. A. Insights into Protein–Protein Binding by Binding Free Energy Calculation and Free Energy Decomposition for the Ras–Raf and Ras–RalGDS Complexes. *J. Mol. Biol.* **2003**, *330* (4), 891-913.
(58) Shapovalov, Maxim V.; Dunbrack, Roland L. A Smoothed Backbone-Dependent Rotamer Library for Proteins Derived from Adaptive Kernel Density Estimates and Regressions. *Struct.* **2011**, *19* (6), 844-858.

(59) Pettersen, E. F.; Goddard, T. D.; Huang, C. C.; Meng, E. C.; Couch, G. S.; Croll, T. I.; Morris, J. H.; Ferrin, T. E. UCSF ChimeraX: Structure visualization for researchers, educators, and developers. *Prot. Sci.* **2021**, *30* (1), 70-82.
(60) Bryksin, A. V.; Matsumura, I. Overlap extension PCR cloning: a simple and reliable way to create recombinant plasmids. *BioTechniques*. **2010**, *48*(6), 463–465.
